# Monitoring and predicting corn grain quality on the transport and post-harvest operations in storage units using sensors and machine learning models

**DOI:** 10.1038/s41598-024-56879-5

**Published:** 2024-03-14

**Authors:** Dágila Melo Rodrigues, Paulo Carteri Coradi, Larissa Pereira Ribeiro Teodoro, Paulo Eduardo Teodoro, Rosana dos Santos Moraes, Marisa Menezes Leal

**Affiliations:** 1 Laboratory of Postharvest, Campus Cachoeira do Sul, Federal University of Santa Maria, Highway Taufik Germano, 3013, Passo DAreia, Cachoeira do Sul, Rio Grande do Sul 96506-322 Brazil; 2 Department Agricultural Engineering, Rural Sciences Center, Federal University of Santa Maria, Avenue Roraima, 1000, Camobi, Santa Maria, Rio Grande do Sul 97105-900 Brazil; 3 Department of Agronomy, Campus de Chapadão do Sul, Federal University of Mato Grosso do Sul, Chapadão do Sul, Mato Grosso do Sul 79560-000 Brazil

**Keywords:** Artificial intelligence, Grain quality, Loss reduction and grain conservation, Post-harvest technologies, Predictive models, Information technology, Scientific data, Environmental sciences

## Abstract

Monitoring the intergranular variables of corn grain mass during the transportation, drying, and storage stages it possible to predict and avoid potential grain quality losses. For monitoring the grain mass along the transport, a probe system with temperature, relative humidity, and carbon dioxide sensors was developed to determine the equilibrium moisture content and the respiration of the grain mass. These same variables were monitored during storage. At drying process, the drying air and grain mass temperatures, as well as the relative humidity, were monitored. For the prediction of the physical and physical–chemical quality of the grains, the results obtained from the monitoring were used as input data for the multiple linear regression, artificial neural networks, decision tree, and random forest models. A Pearson correlation was applied to verify the relationship between the monitored and predicted variables. From the results obtained, we verified that the intergranular relative humidity altered the equilibrium moisture content of the grains, contributing to the increased respiration and hence dry matter losses along the transport. At this stage, the artificial neural network model was the most indicated to predict the electrical conductivity, apparent specific mass, and germination. The random forest model satisfactorily estimated the dry matter loss. During drying, the air temperature caused volumetric contraction and thermal damage to the grains, increasing the electric conductivity index. Artificial neural network and random forest models were the most suitable for predicting the quality of dry grains. During storage, the environmental conditions altered the moisture contents causing a reduction in the apparent specific mass, germination, and crude protein, crude fiber, and fat contents. Artificial neural network and random forest were the best predictors of moisture content and germination. However, the random forest model was the best predictor of apparent specific mass, electrical conductivity, and starch content of stored grains.

## Introduction

The increase in grain production in crops has been occurring year after year due to the application of new technological packages, mainly focused on precision agriculture to optimize the application of inputs, machinery, and the use of natural resources in order to improve agricultural processes and hence increased grain yields^1^. Thus, all the investment made in farming is expected to be converted into post-harvest grain yield^2^. Besides higher grain production, technologies that provide grain storage with quality for marketing and processing are required^3^ to ensure higher profits for the farmer and industry^4^.

Post-harvest consists of different stages and processes that are at the end of the grain production chain^5^, where they also influence the sector's logistics^6^ through transportation and grain storage^7^. Losses in these steps can occur by grain metabolic changes influenced by environmental conditions, process actions, and product movement^7^.

After harvesting, the grain mass with high impurity and moisture contents can be transported for long distances, remaining stored in the vehicle bodies without any control of the qualitative alterations that can occur due to temperature variations, relative humidity of the intergranular air^8^. The transfer of heat and humidity between the grains and the intergranular air can elevate the grain mass temperature and increase the product respiration, causing dry matter consumption and physical and physicochemical alterations of the grains. Often, the initial levels of deterioration of the grain mass during transportation are not immediately noticeable, and are aggravated throughout the drying and storage processes^9^.

In drying, the high temperature and the flow of the grain mass in the dryer are the main factors influencing the product quality. Thus, the control of the drying air and grain mass temperature, as well as the drying time on the initial and final moisture content of the product should be monitored to avoid losses^10^. The damage in the cellular tissues caused by drying adds to the deteriorations from the previous stages, aggravating even more in the subsequent stage, when the grains are stored inadequately^11^. At storage, even under safe conditions in terms of moisture content in the grains, the way, conditions and time of storage of the batches can cause heating and elevate the grain respiration rates^12^.

Advances in data acquisition and processing techniques have been applied with global success to aid decision making in different agricultural processes^13^. The use of crop sensors has become increasingly common in pre-harvest grain yield estimation^14^, nutritional status and weed monitoring^15^, and determination of rates of cover nitrogen fertilization, water stress^16^ and grain protein content^17^. Technologies that assist in the estimation of the nutritional state, grain production and quality contribute to greater efficiency in the application of inputs, thus reducing spending on unnecessary inputs and decreasing environmental impacts^10^.

Using sensors associated with the Internet of Things and Artificial Intelligence can assist in monitoring and predicting the quality of grains in post-harvest processes. The application of these tools can support the control of post-harvest processes by using a set of advanced information, communication, analysis, and data processing techniques, such as Big Data analysis and digital platforms that allow extracting a large amount of information about the collected data for decision making^10,18^.

Therefore, the determination of the equilibrium moisture content of the grain mass by measuring the temperature, the relative humidity of the intergranular air and the moisture content of the grains in the different post-harvest steps can make it possible to control the intensity of deterioration and avoid the loss of grain quality^11^. Whereas measuring the carbon dioxide concentration in the intergranular air or in the environment that the grain mass is in can provide an early response to the respiratory intensity of the grain.

These monitored variables are used as input data for predicting grain mass quality through Machine Learning (ML) models^19^. Random Forests (FA) is an ML technique successfully used in yield prediction and grain quality assessment^20^. This model has proven to be efficient and easier to use for predicting corn and wheat quality when compared to multiple linear regression models^21^. Artificial Neural Networks (ANN) are another method that can be trained from data related to corresponding inputs and outputs^22^. ANNs are useful tools for analyzing and interpreting complex food safety data, predicting the physical and chemical quality of grains^23^. In this sense, machine learning models have been widely used to predict the quality of soybeans during transport^21^ and stored corn^18^, in determining the quality of wheat during storage^24^, as well as in the evaluation of the germination rate of stored soybean seeds^25^. Some recent studies have demonstrated the effectiveness of machine learning models in predicting the viability, vigor and germination speed of seeds of different crops. Lin et al.^26^ obtained satisfactory results using machine learning algorithms; however, the models that best predicted soybean quality varied depending on processing and storage conditions.

Thus, the real-time monitoring of intergranular variables of the grain mass, in order to preserve the quality of the product and reduce as much as possible the losses in the different stages of post-harvest, makes it possible to indirectly evaluate the potential physical and technological changes of the grains using predictive algorithms^20^. In this context, the application of ML models can accurately predict the possible grain quality losses through easily measured variables^10^. Thus, the objective of this study was to predict corn grain quality at the transportation, drying, and storage stages by real-time monitoring of easily measured intergranular variables using sensors and ML models.

## Material and methods

### Experimental characterization

**Figure 1:**
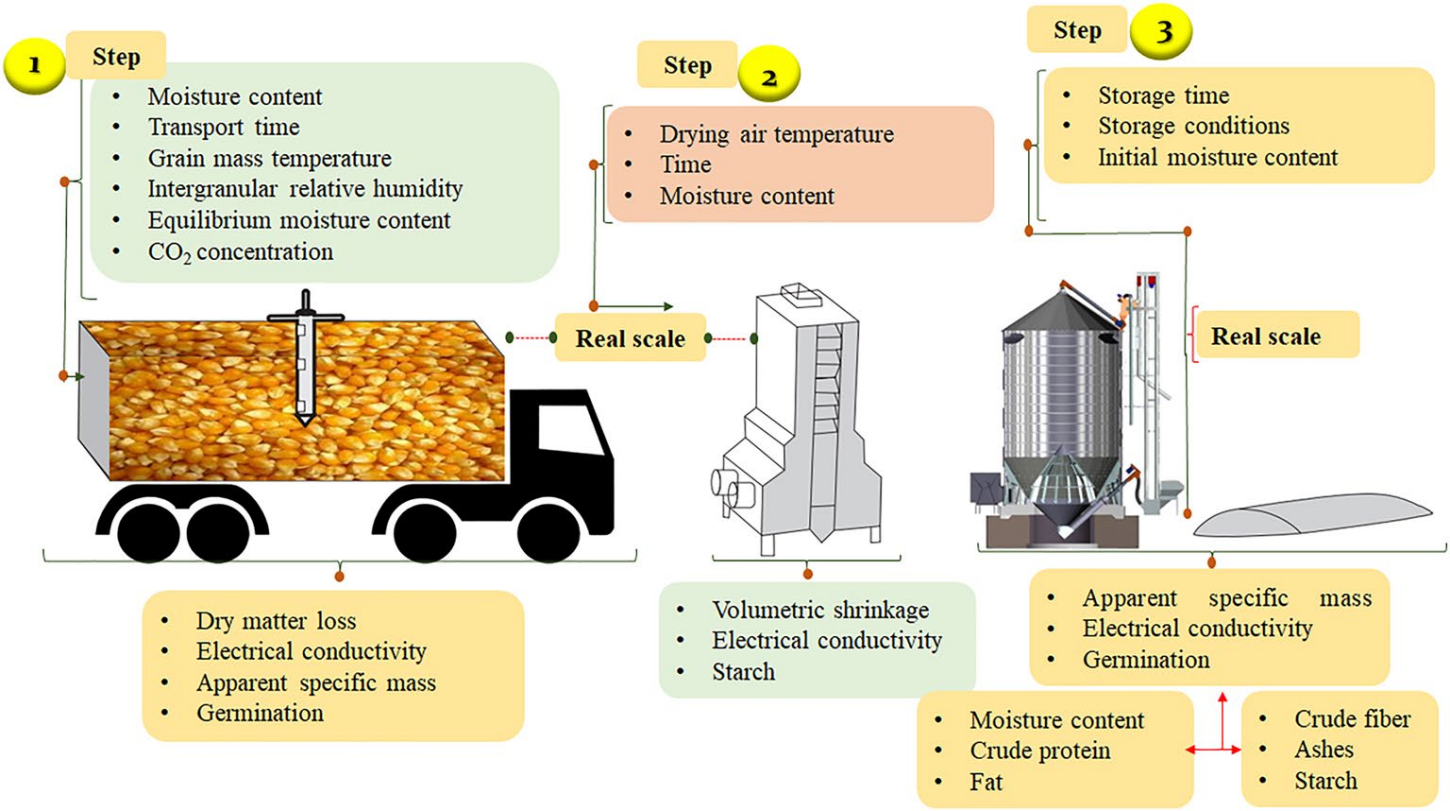
Experimental characterization in the post-harvest, transportation, drying, and storage stages of corn grains.

The experiment was carried out on a real scale in commercial storage units involving the transportation, drying and storage steps of corn grains (Fig. 1). The data collection for each step was performed through indirect monitoring of the corn grain quality, using technologies developed in the laboratory.

### Technologies used for monitoring corn grain mass

**Figure 2:**
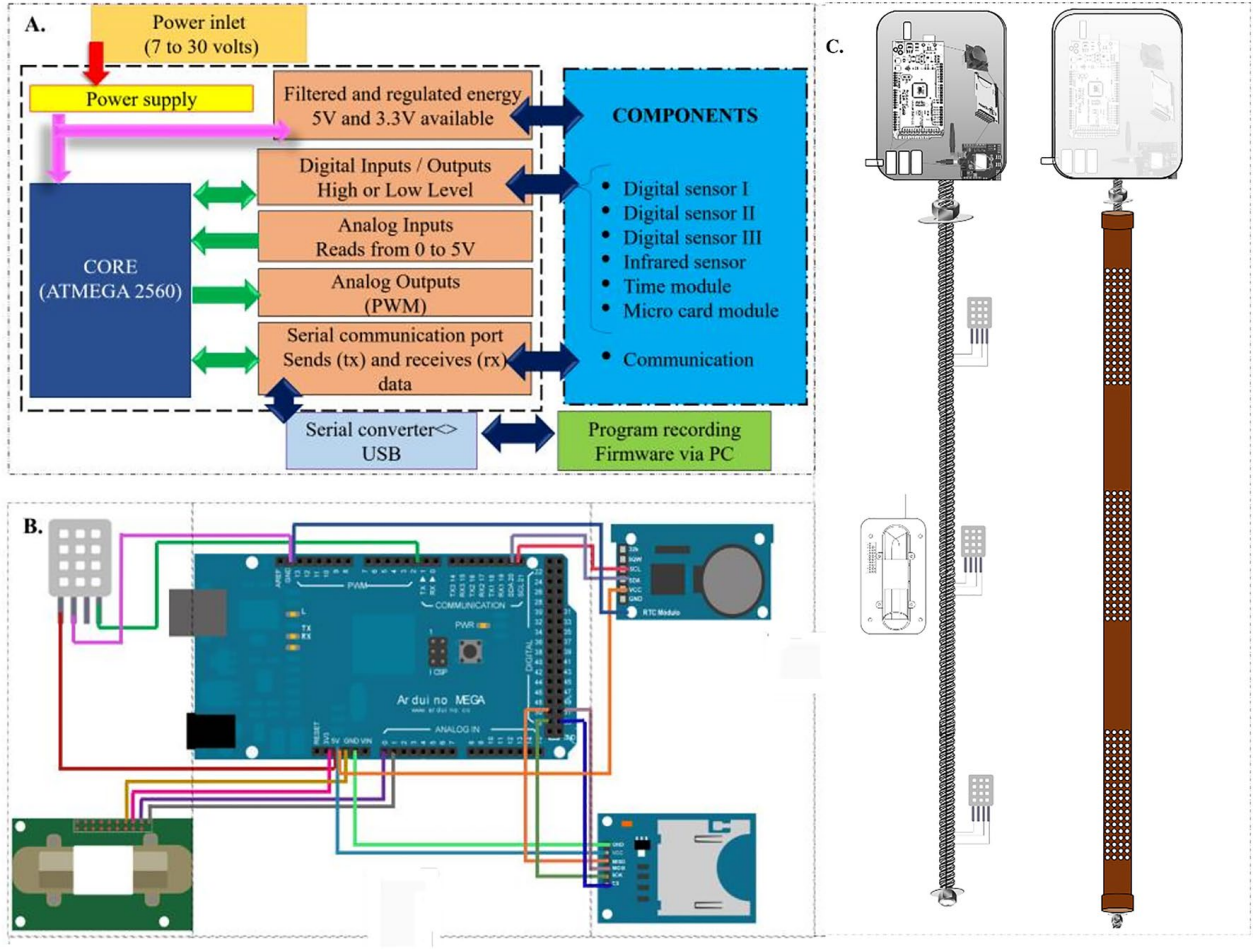
Block diagram of the components of the device control system (**A**), micro-controller connection by jumper cables (**B**), and conditioning and attachment of the set of sensors to the polyvinyl chloride probe (**C**).

For monitoring the corn grain mass, a portable device has been developed. The device consists of an Arduino Mega 2560 microcontroller (model Mega 2560, Arduino LLC, Italy) as the control core. The system hardware includes three digital sensors to detect air temperature and relative humidity (model DHT22, Aosong Electronics, Guangzhou, China), a non-destructive infrared sensor to detect CO_2_ concentration (model MHZ-14, Winsen, China), real-time clock modules (model DS3231, flip-flop, China), and a micro-SD card (model Greatzt micro SD card, import, China). A control system block diagram is shown in Fig. 2A. Output data from the digital sensor, infrared sensor, and modules are connected to the microcontroller's I /O communication terminals responsible for physical communication and component integration, and data calculation. A schematic of connecting each component via jumper cables is shown in Fig. 2B^27^.

The temperature and relative humidity sensors (model DHT22, Aosong Electronics, Guangzhou, China) were attached to three ends of a threaded bar and the CO_2_ sensor (model MHZ-14, Winsen, China) was attached to the central part. The real-time clock module (model DS3231, flip-flop, China) and the micro-SD card (model Greatzt micro-SD card, Import, China) were stored in a box. Figure 2C shows the structure of the device with the sensors arranged along the threaded bar and protected by a polyvinyl chloride probe. The device has a power supply with three batteries arranged in series, and the total power is 27 V^27^.

**Figure 3:**
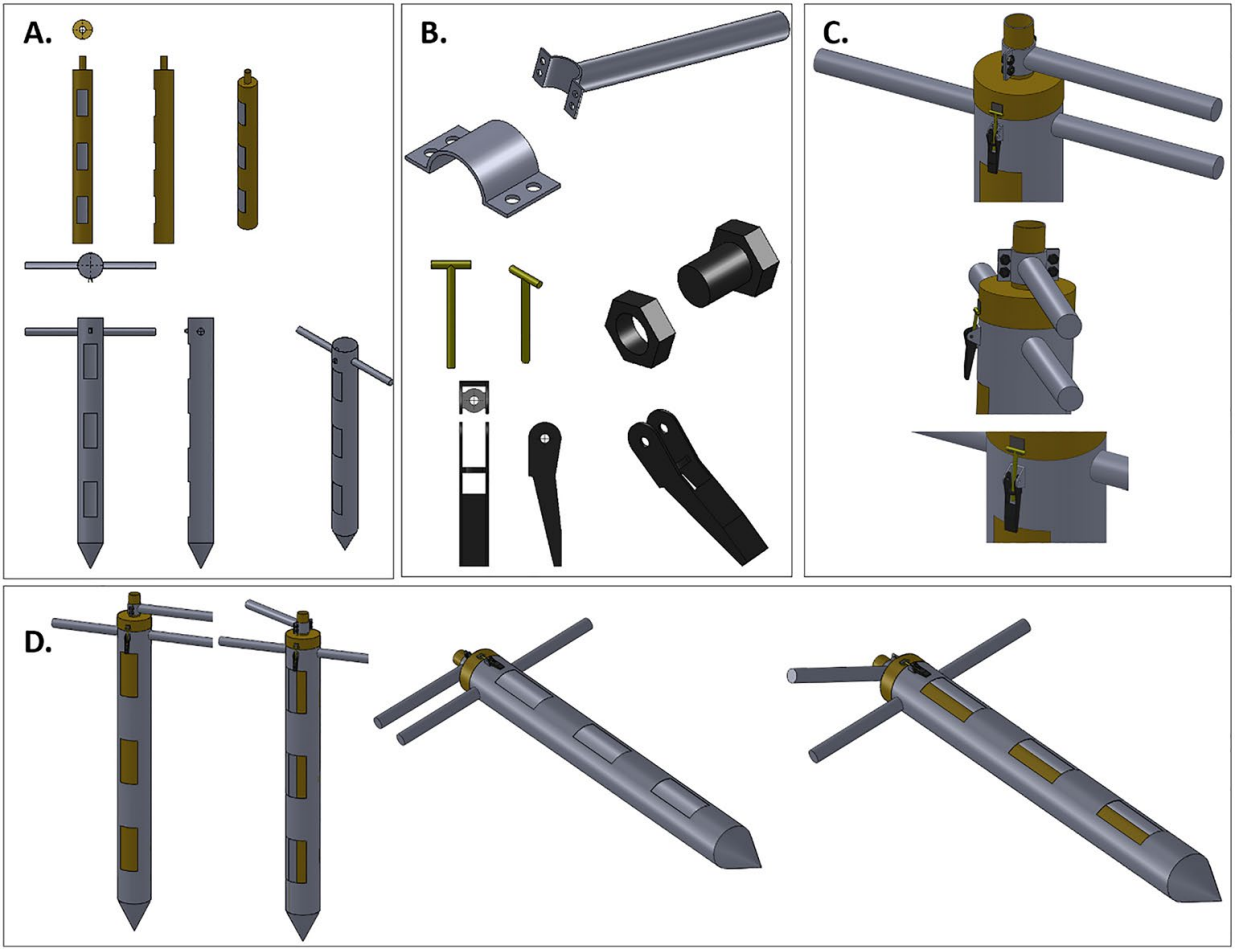
Grain sampling tube for conditioning the probe. (**A**) internal and external view of the tube, (B) parts for sealing, (**C**) top view of the sampler tube, (**D**) complete view of the tube, (**E**) application of the monitoring system in the corn grain mass.

The software used on the Arduino board was programmed based on the C +  + programming language, with most of the libraries provided by the platform^28^. The Arduino IDE (Integrated Development Environment) was used to develop the embedded firmware for the Atmega 2560 microcontrollers^29^. A metal grain sampling tube was designed to couple the probe. The tube consisted of two overlapping tubes, with a tip at the bottom and a swivel arm at the top. This enabled the probe to be protected and increased the accuracy of the intergranular grain reading. The tube contained openings/cells at the top, middle, and bottom along its length, as shown in Fig. 3^27^.

### Monitoring the grain mass in transport

**Figure 4:**
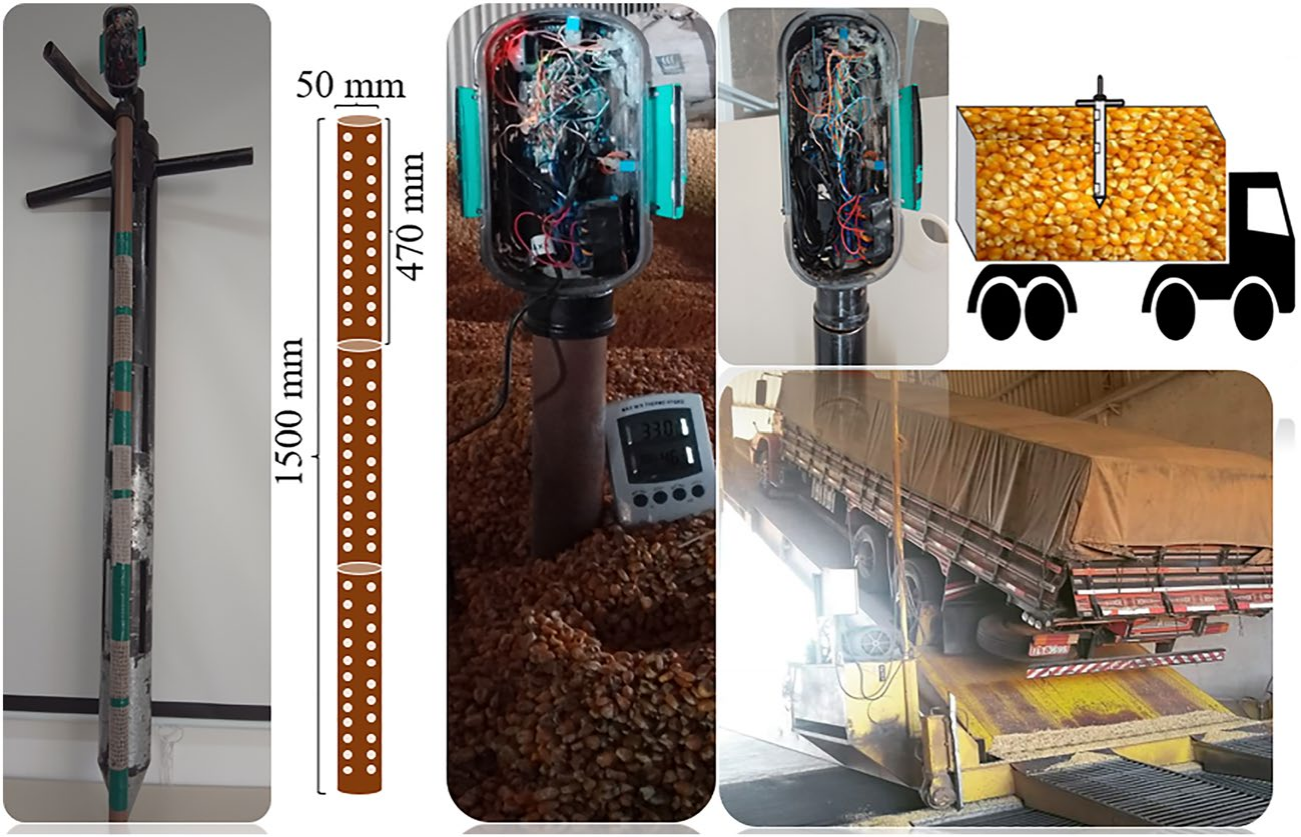
Experimental characterization of data collection at the corn grain transport stage.

Metallic sampler and non-destructive probe with sensors were inserted in the grain mass to measure the variables temperature, relative humidity and carbon dioxide in the porosity, in real-time, at intervals of 1.87 s for 24 h of transport with grains at 11, 14 and 18% moisture (Fig. 4). Grain sampling was performed at 0, 120, 480, and 1440 min of transport at three positions in the grain mass profile (top, middle, and bottom) of the load. With the results obtained from the monitoring, the equilibrium moisture content (EMC) of the grain mass was determined, and the dry matter loss (DML) was calculated. Furthermore, the monitored variables were adopted as input data in the machine learning models for grain quality prediction. For each sample collected, grain quality assessments such as apparent specific mass (ASM), electrical conductivity (EC), and germination (GERM) were performed and used for predicting corn grain quality.

### Monitoring the grain mass during drying

**Figure 5:**
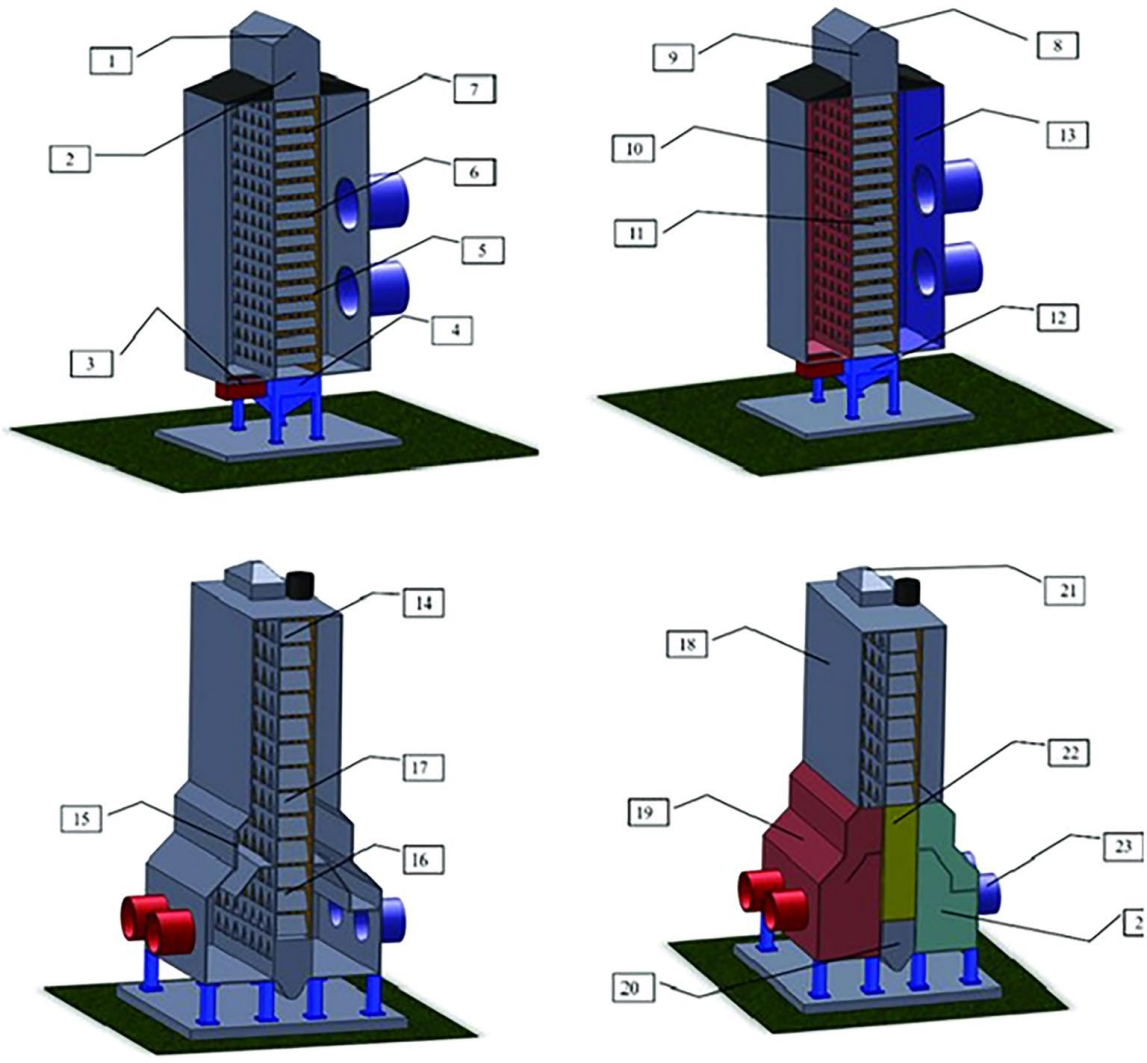
Grain Dryers: 1—Maximum level sensor, 2—Minimum level sensor, 3—Hot air inlet sensor, 4—Stop sensor, 5—Mass sensor, 6—Mass sensor, 7—Mass sensor, 8—Input product, 9—Load box, 10—Drying chamber, 11- Drying tower, 12—Discharge table, 13—Drying air, 14—Maximum level sensor, 15—Hot air inlet sensor, 16—Grounding sensor, 17—Minimum level sensor, 18—Equalization chamber, 19—Hot air chamber, 20—Discharge table, 21—Product inlet, 22—Drying tower, 23—Fans, 24—Cold air chamber.

The corn grains were harvested with 18% moisture content. Then, impurities and foreign matter were removed using an air machine and sieve. Afterwards, the grains were subjected to drying in a mixed-flow continuous dryer with a nominal capacity of 80 ton h^-1^ and drying air temperature of 80, 100 and 120 °C (Fig. 5). Three drying tests were performed, and during the tests, samples of 10 in 10 min at the bottom of the dryer (outlet) were collected for determination of moisture content (WC), as well samples to determine the volumetric shrinkage (VS), electrical conductivity (EC), and starch yield (STA). The drying was performed until the grains reached 12% moisture content. During the drying, the grain mass temperature was monitored by using thermocouple sensors installed in the dryer itself at the drying chamber. The temperature and air relative humidity were monitored during the process.

### Monitoring grain mass in storage

**Figure 6:**
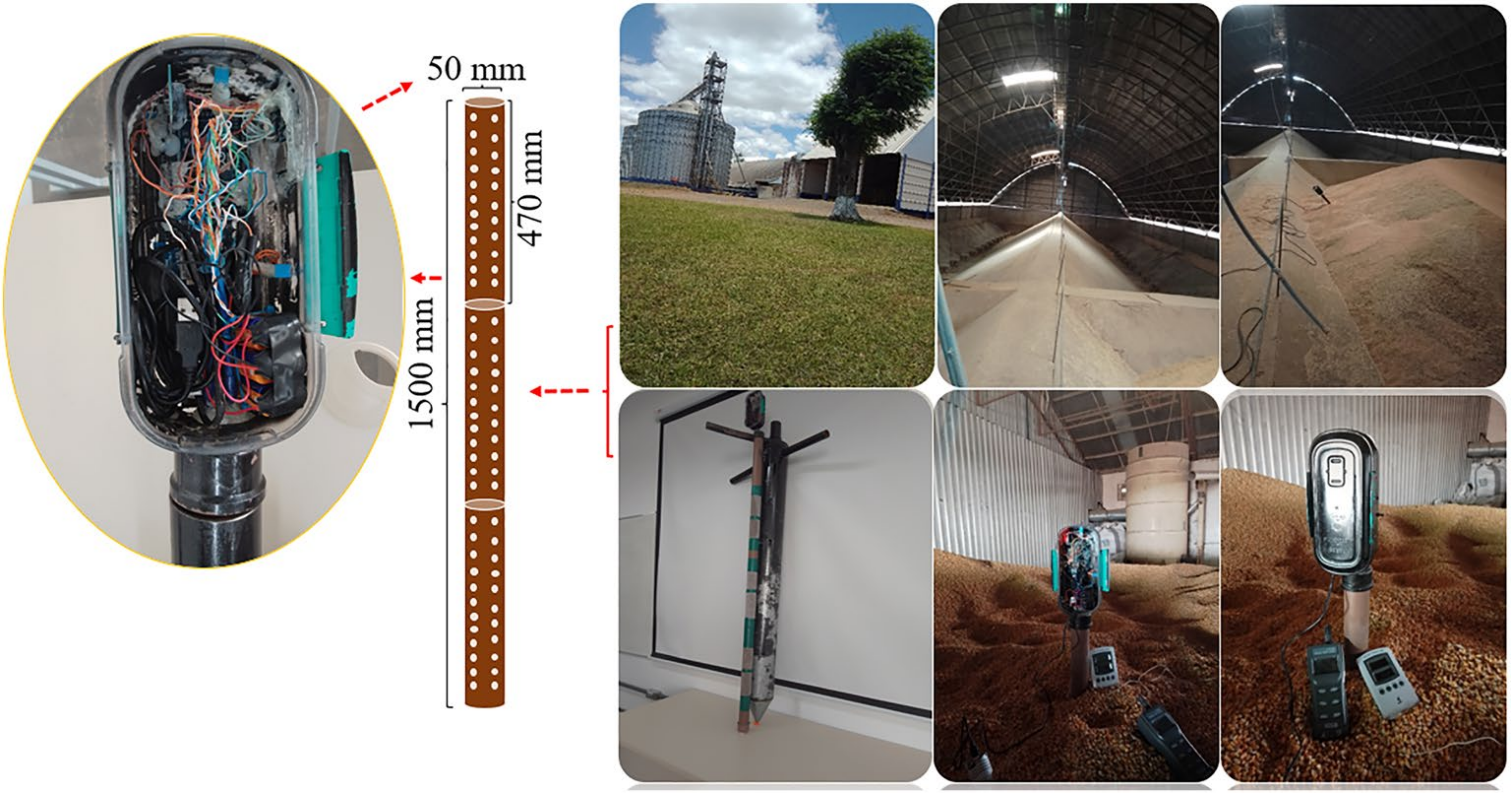
Experimental characterization of data collection at the corn grain storage stage.

A mass of dried corn grains with 12% moisture content was stored in metal silos over six months (Fig. 6). During the three-month storage period, the temperature and relative humidity of the intergranular air were monitored to determine the equilibrium moisture content and the carbon dioxide (CO_2_) concentration to obtain the early dry matter loss over a 20-h period. With the results obtained from the monitoring, a prediction of the quality of the stored corn kernels was made.

### Evaluations of corn grain quality

The calculations of the equilibrium moisture content of the grain mass were performed by Eqs. (1) (0 < RH < 55) and (2) (55 < RH < 100)^30^:

1 $$EMC=\frac{3.96{RH}^{0.492}}{{\text{ln}}(T)}$$

2 $$EMC=\frac{6.21{\text{exp}}(0.0274RH)}{{\text{ln}}(T)}$$

em que, EMC: Equilibrium moisture content (%, d.b.), RH: Relative humidity (%), T: Temperature (°F).

Dry matter loss was calculated by the monitored CO_2_ concentration in the corn grain mass, using Eq. (3)^31^:

3 $${\text{DML}}=100\left({{\text{C}}}_{{{\text{CO}}}_{2}}- \Delta {{\text{C}}}_{{{\text{O}}}_{2 }}\right)\left(\frac{\upvarepsilon {{\text{PW}}}_{{\text{g}}}}{2{\text{ASM}}\left(1-{\text{MC}}\right){\text{RT}}}\right)$$

wherein, DML: Dry matter loss (%), ${{\text{C}}}_{{{\text{CO}}}_{2}}$: ${{\text{C}}}_{{{\text{CO}}}_{2}}$ concentration (v/v) measured inside the metal silos, $\Delta {{\text{C}}}_{{{\text{O}}}_{2}}$: change in ${{\text{O}}}_{2}$ concentration throughout storage considering the initial concentration of 21%, $\upvarepsilon$: porosity of the granular mass (40%), P: pressão atmosférica local (96 kPa), W_g_: molar mass of glucose (180 kg kmol^−1^), ASM: apparent specific mass of the grains (kg m^−3^) (750 kg m^−3^), MC: moisture content of the grains (decimal, d.b.), R: perfect gas constant (8,314 kJ Kmol^−1^ K^−1^), T: Temperature (K).

Moisture content of the grains (%) was determined by the gravimetric method (analytical balance 0.0001, model AUY-220-I)^26^. The volumetric contraction of the grains was determined by Eq. (4), in which the major, medium and minor axis of one hundred grains for each sample were measured using a digital pachymeter.

4 $${\text{VS}}=\frac{\pi abc}{6}$$

wherein: VS: volumetric shrinkage (mm^3^), a: main grain axis (mm), b: middle grain axis (mm), c: minor grain axis (mm).

The electrical conductivity test was performed with fifty grains in three subsamples of each treatment, weighed with precision to two decimal places (0.01 g). The samples were placed to soak in plastic cups with 75 mL of deionized water and kept in a refrigerated chamber with controlled temperature at 25 ± 2 °C for 24 h. The solutions containing the grains were slightly shaken to homogenize the leachates and immediately read in a portable conductivity meter CD-850 "Instrutherm", with the results divided by the mass of 25 grains and expressed in µS cm^−1^ g^−1^ of grains^32^.

For the germination test, four subsamples of 50 seeds from each experimental unit were used, distributed on paper towel rolls (Germitest), and moistened with distilled water in an amount 2.5 times the mass of dry paper. Then, the rolls with the seeds were placed in a germinator (Mangesdorf), regulated at 25 °C ± 2 °C. The evaluations were performed on the eighth day after the test installation, counting normal and abnormal seedlings and dead seeds according to criteria established in the Rules for Seed Analysis^32^.

To determine starch (STA), crude protein (CP), fat (FAT) and ash (ASH) in corn grains, near-infrared spectroscopy (NIRS) (Metrohm, DS2500 spectrometer, Herisau, Switzerland) with high optical accuracy was used. The samples were homogenized and placed in the sampling dish. The analysis was based on illuminating a sample with radiation of a specific wavelength in the near-infrared and then measuring the difference between the energy emitted by the spectroscope and reflected by the sample to the detector. This difference was measured in several bands, creating a spectrum for each sample. The result obtained was compared to a calibration set.

### Correlation analysis

A Pearson correlation network between the monitored and predicted variables was generated. These analyses were performed using the Rbio software, following the procedures recommended by Bhering et al.^33^.

### Machine learning analysis

**Figure 7:**
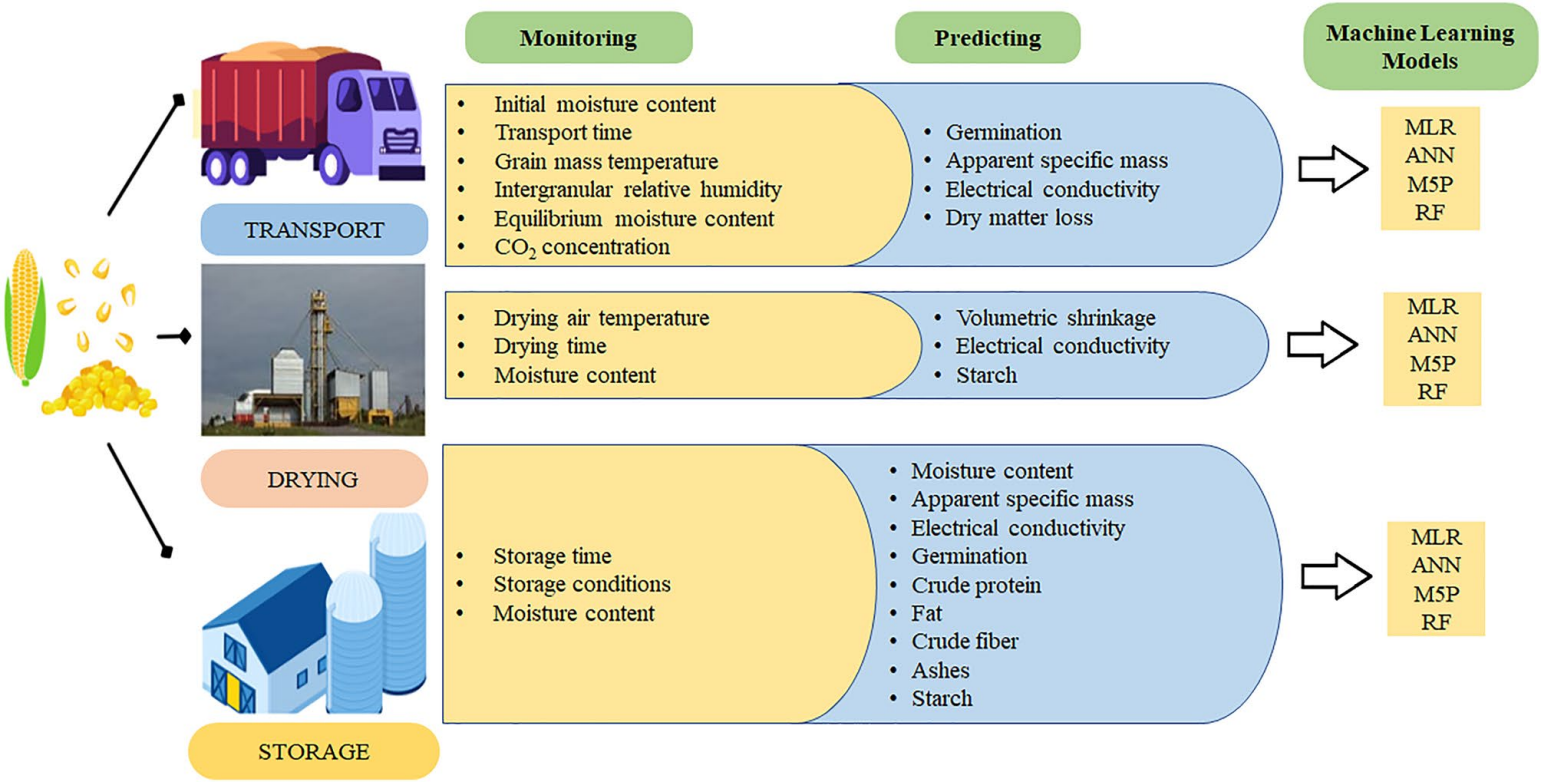
Experimental characterization of the applicability of Machine Learning models on monitored and predicted variables in the transportation, drying and storage stages of corn grains.

Data were analyzed on Weka software version 3.9.5. testing the following models: multiple linear regression (MLR), artificial neural network (ANN), Quinlan's M5 algorithm (M5P) and random forest (RF) (Fig. 7). The RLM model was used as a control model. The ANN tested consists of Multilayer Perceptron with a single hidden layer, whose number of neurons is equal to the number of attributes plus the number of classes, all divided by 2^34^. The tested M5P model is a reconstruction of Quinlan's M5 algorithm that is based on the conventional decision tree with the addition of a linear regression function to the leaf nodes^35^. The RF model is able to produce multiple prediction trees for the same dataset and use a voting scheme among all these learned trees to predict new values^36^. The ML analyses were performed using the default software setting for all models tested^37^ on an Intel^®^CoreTM i5-3317U CPU with 4 Gb RAM.

Prediction of moisture content, apparent specific mass, dry matter loss, electrical conductivity, germination, volume shrinkage, starch yield, crude protein, fat and ashes contents in corn grains was performed using MLR, ANN, M5P and RF models with stratified cross-validation with ten folds (k-fold = 10) and ten repetitions (runs). Different inputs were used for each model to predict the quality variables. For transportation, moisture content, time, temperature, relative humidity, and equilibrium moisture content were used. For drying, drying air temperature, drying time, and moisture content were used. For storage, storage time, storage conditions, and grain moisture content were used (Tables S1, S2, and S3).

### Statistical analysis

For the three post-harvest steps (transportation, drying, and storage), the correlation coefficient (r) and the mean apparent error (MAE) were obtained to analyze the prediction accuracy of the models. Next, variance analysis was performed adopting the completely randomized design, in which the ML models (ANN, M5P, and RF) and the multiple linear regression (MLR) were compared. Ten repetitions (folds) were adopted for each model. For comparison of the models, MAE and r means for each model were grouped by the Scott-Knott test at 5% probability and shown through boxplot graphs. These analyses were performed on the R software using the ExpDes.pt and ggplot2 packages.

### Ethics approval

The authors declare that the research was conducted within ethical standards and that there is no ethical conflict to highlight in this work.

### Consent to participate

The authors declare consent to participate in the research.

## Results and discussion

### Monitoring and predicting the quality of corn grains during transportation

**Figure 8:**
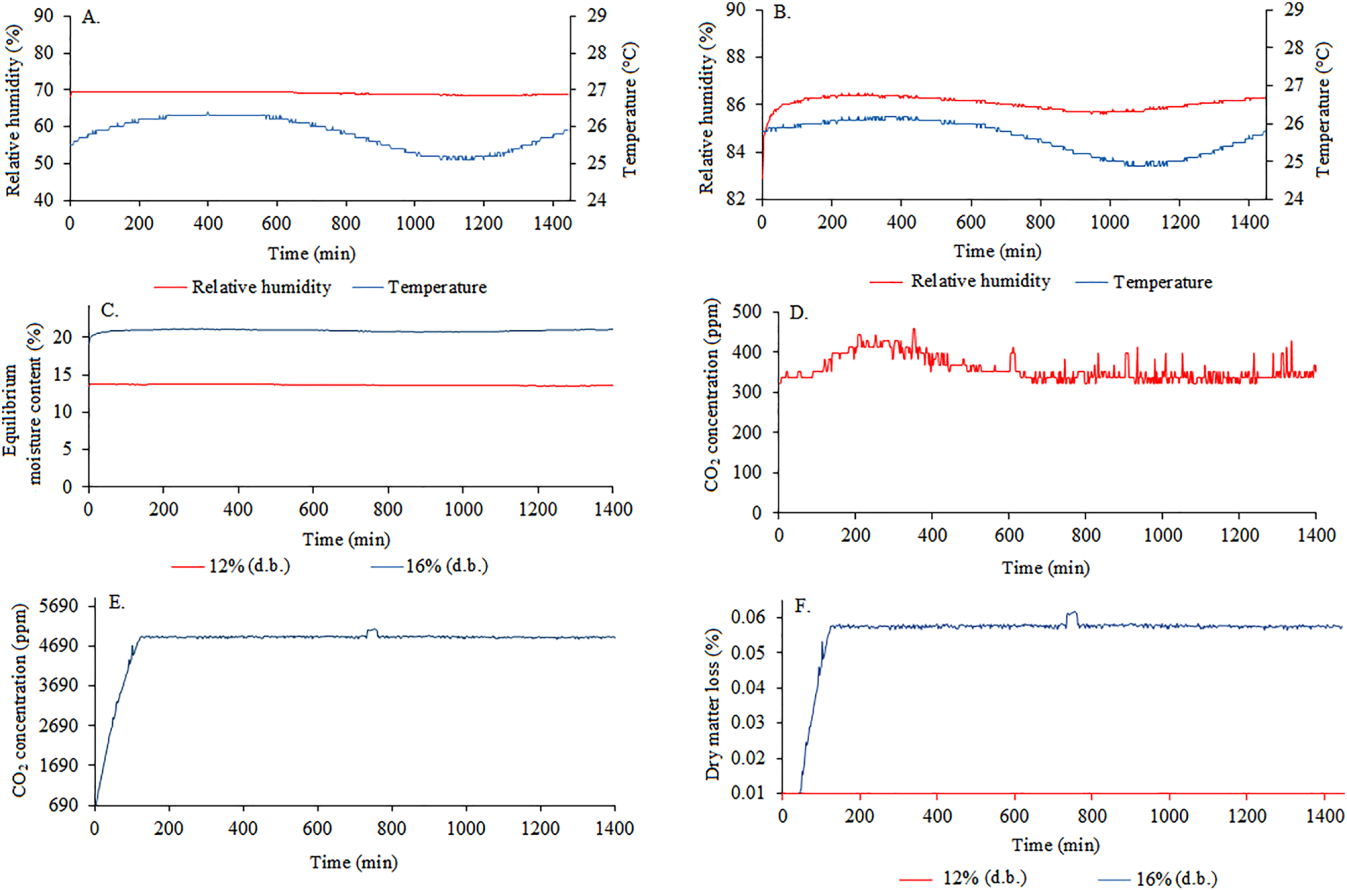
Monitoring relative humidity and intergranular air temperature at 12% (**A**) and 16% (**B**) moisture content, equilibrium moisture content at 12% and 16% moisture content (**C**), carbon dioxide concentrations at 12% (**D**) and 16% (**E**) moisture content, and dry matter loss (**F**) at 12% and 16% moisture content in corn grains throughout transportation.

When monitoring grains with 12% moisture content (Figs. 8A), the intergranular relative humidity remained constant and close to 70%. However, the intergranular air temperature oscillated throughout the monitoring time. For grains with 16% moisutre content (Fig. 8B), the intergranular temperature remained close to the conditions of 12% moisture content, while the intergranular relative humidity was above 86%. In addition, the equilibrium moisture content was found to rise to 20% and remained constant (Fig. 8C). The intergranular variables indicated possible cellular respiration, which in turn raised carbon dioxide (CO_2_) levels in the grain mass throughout the transport period, evidencing that the grain metabolism was active with high respiratory activity^38^. We point out that grain mass, when entering equilibrium moisture content with moisture contents above 12%, may indicate deterioration risks if travel time in transportation is prolonged, resulting in corn grain quality losses^39^.

At 12% moisture content conditions (Fig. 8D), the grain mass did not have marked respiration, remaining below and close to acceptable natural environment levels of 420 ppm. However, at 16% moisture content (Fig. 8E), carbon dioxide (CO_2_) levels were high, reaching 4960 ppm, indicating a high respiration intensity of the grains with high deterioration risks^40^. At 16% moisture content, there was an increase in intergranular relative humidity and heating of the corn grain mass, becoming metabolically active. With the heating of the grain mass, mass and heat transfer and grain cell respiration increased^41,42^.

In Fig. 8F, it can be seen that the grains with moisture contents at 12% did not alter the dry matter consumption of the grains, agreeing with the results monitored in Fig. 8A and D. However, in the grains with 16% moisture contents, there were dry matter losses close to 0.06% over the twenty-hour monitoring period. This combination resulted in higher enzymatic and biological activities, favoring the development of insects and infection by fungi and bacteria, triggering reactions of degradation of the quality of the grains^43^.

**Figure 9:**
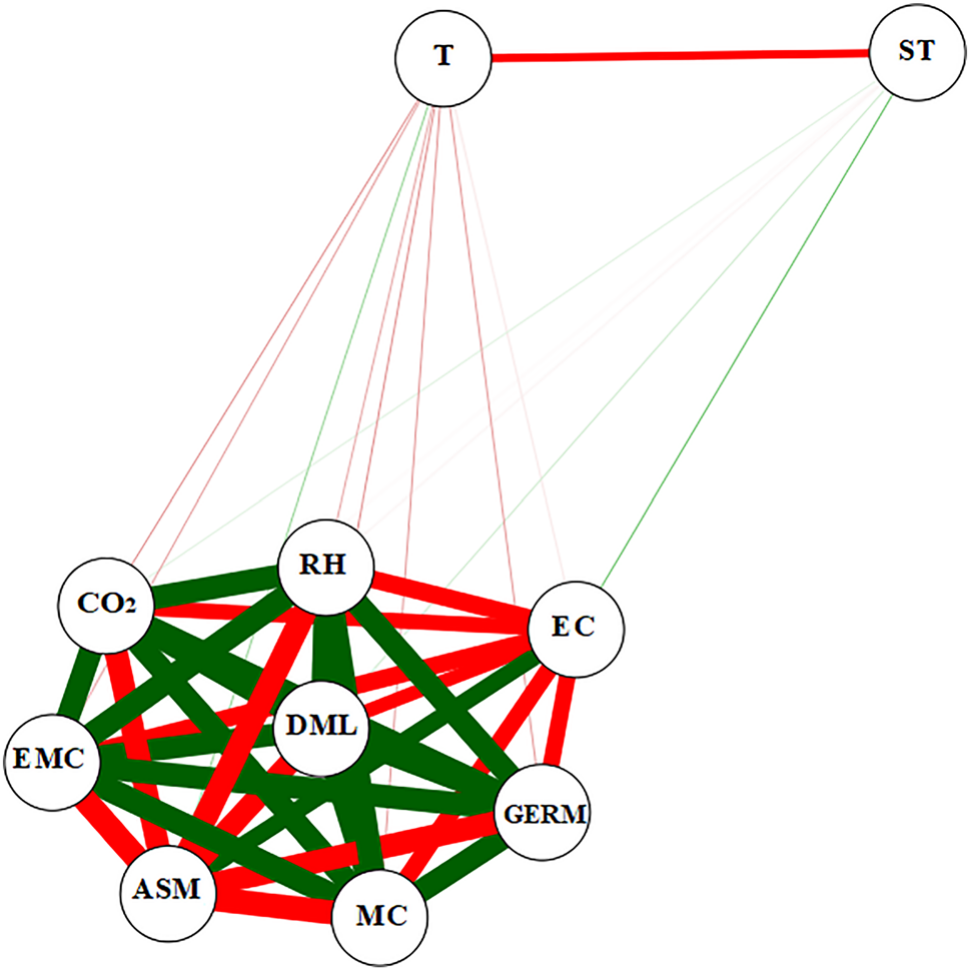
Pearson correlation network between the variables apparent specific mass loss (ASM), electrical conductivity (EC), germination (GERM), dry mass loss (DML), moisture content (MC), time (ST), temperature (T), relative humidity (RH), equilibrium moisture content (EMC), and carbon dioxide concentrations (CO_2_) at the transport stage.

Pearson's correlation network (Fig. 9) indicated a relationship of the monitored variables with grain quality as a function of 12 and 16% moisture contents. There was a positive and strong correlation of dry matter loss (DML) with relative humidity (RH), moisture content (MC), equilibrium moisture content (EMC), and carbon dioxide (CO_2_), and strongly negative with apparent specific mass (ASMThese results are consistent with Fig. 8A–E for 12% and 16% grain moisture contents. Germination analysis (GERM) obtained a positive and strong correlation with moisture content (MC), intergranular relative humidity (RH), and dry matter loss (DML). However, there was a negative correlation between these variables with apparent specific mass (ASM) and electrical conductivity (EC). The GERM was directly dependent on the intergranular relative humidity conditions, as well as the moisture levels with the metabolic activity of the grains.

The correlation of MC and RH variables affected dry matter and biochemical properties, inhibiting the components that conferred grain germination. The variable EC had a medium positive correlation with ASM and negative with RH, EMC and DML. There was negative correlation between time (ST) and temperature (T), CO_2_, EMC, RH, DML, MC, GERM, and EC, indicating that increased transport time provided higher changes in grain quality.

**Figure 10:**
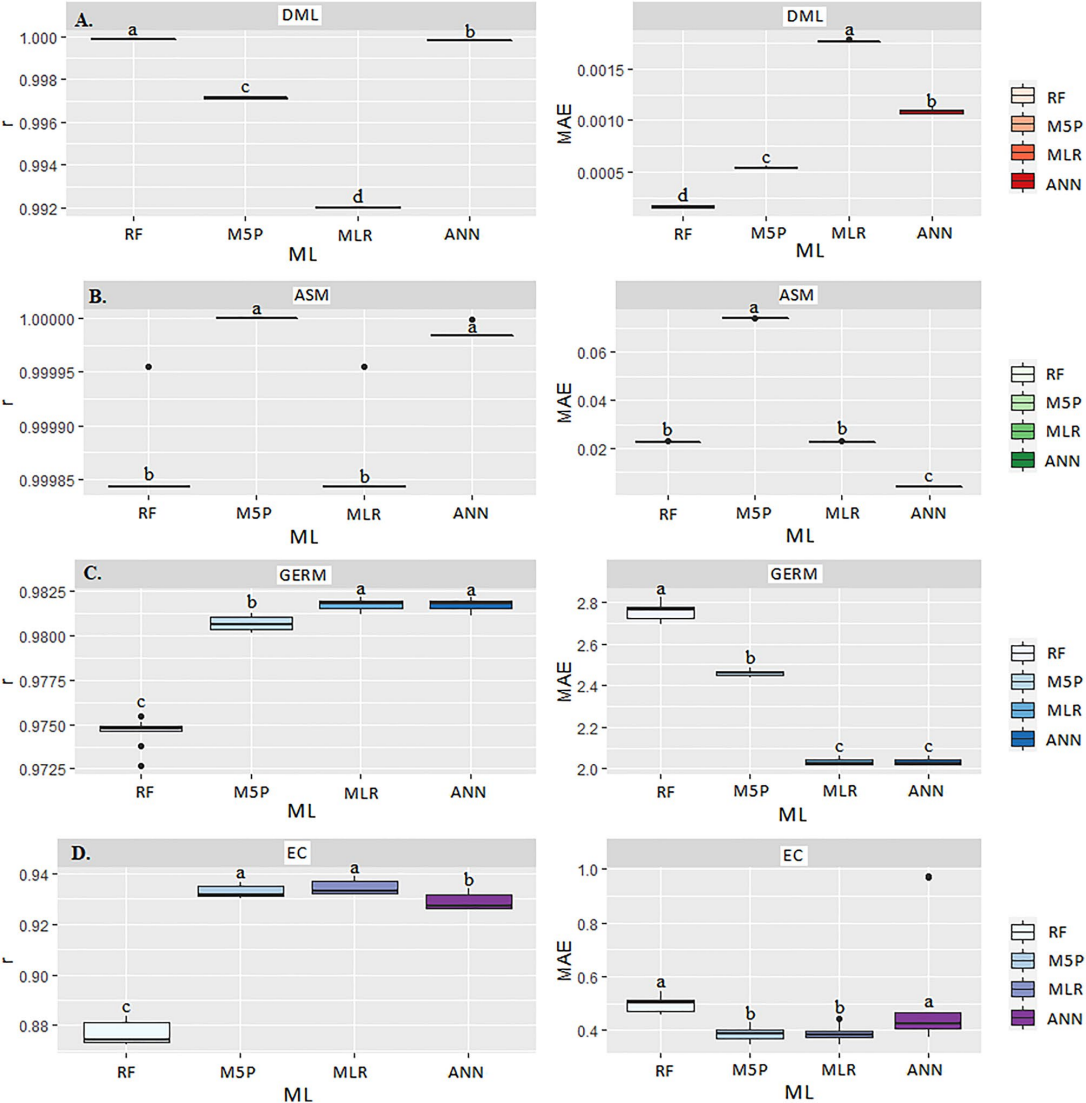
Boxplot for mean comparison of correlation coefficient (r) and mean absolute error (MAE) between the multiple linear regression (MLR) and Machine Learning models: Artificial Neural Networks (ANN), Quinlan's M5 Algorithm (M5P), and Random Forest (RF) for predicting dry matter loss-DML (**A**), apparent specific mass-ASM (**B**), germination-GERM (**C**), and electrical conductivity-EC (**D**), in corn grains at transport stage.

In Table 1 and Fig. 10A are the results of correlation coefficients (r), coefficients of determination (R^2^), and the mean absolute error (MAE) of the prediction of corn grain quality during transport: dry mass loss (DML), apparent specific mass (ASM), germination (GERM), and electrical conductivity (EC). Considering the different Machine Learning (ML) models and input variables in the models (moisture content, transport time, temperature, relative humidity, equilibrium moisture content, and carbon dioxide concentration), significance was observed at p < 0.05 by the Scott Knott (SK) test for the quality variables.

**Table 1:** Correlation coefficient (r), mean absolute error (MAE), and coefficient of determination (R^2^) between the observed and estimated values of dry matter loss, apparent mass specific, germination, and electrical conductivity of corn grain in the transport operation for the different Machine Learning models.

Models	r	MAE	R^2^	r	MAE	R^2^
	DML			ASM		
MLR	0.99d	0.00a	98.01	1.00b	0.00b	100.0
ANN	1.00b	0.00b	100.0	1.00a	0.00c	100.0
M5P	0.99c	0.00c	98.01	1.00a	0.07a	100.0
RF	0.99a	0.00d	98.01	1.00b	0.00b	100.0

Models	r	MAE	R^2^	r	MAE	R^2^
	GERM			EC		
MLR	0.98a	2.45c	96.33	0.92a	0.39b	84.64
ANN	0.98a	2.49c	96.19	0.92b	0.39a	84.64
M5P	0.98b	2.98b	96.33	0.93a	0.38b	86.49
RF	0.97c	2.72a	94.88	0.88c	0.50a	77.44

*Equal letters in the column do not differ at p < 0.05 by the Scott Knott test. Dry Matter Loss (DML). Apparent Specific Mass (ASM). Germination (GERM) and Electrical Conductivity (EC). Multiple Linear Regression (MLR). Artificial Neural Networks (ANN). Quinlan's M5 Algorithm (M5P) and Random Forest (RF).

For the variable dry matter loss (DML) of grain mass during transport, we verified that the artificial neural network (ANN) and random forest (RF) models outperformed the other models (Table 1 and Fig. 10B). The grain mass during transport suffered actions of several variants, which are conditioning effects on grain cellular respiration, among them moisture content, temperature, and intergranular relative humidity. The metabolic intensification caused the grain mass to lose part of its dry matter, predicted by the response of the ML models, mainly by RF.

The RF algorithm presented alternatives for prediction, where they randomly chose the conditioning factors, electing a single variable that could interfere most with quality. Compared to other ML models, RF made a faster prediction, as observed in other studies for determining the quality of soybean seeds stored in different packages^44^. Some studies have found that the RF technique performs better for predicting soybean seed dry mass loss in environments with different relative humidity and storage temperature^45^.

For the ASM, the tested Machine Learning (ML) models showed high correlation coefficients, except for the M5P model (Table 1 and Fig. 10). When applied the SK test (p < 0.05), the ANN and M5P models had a better fit, satisfactorily predicting the apparent specific mass (Fig. 10B). During the transport time, the ASM underwent changes, influenced by the variables moisture content and temperature that acted simultaneously on the respiration process.

ASM has been defined as a physical variable that relates the dry grain mass to its total volume^46^. The change in ASM inferred in technical grain breakage and influenced total dry matter, as they are correlated. This event can be verified by the ANN and M5P models, which provided the best results of (r) and (MAE). ANNs were algorithms with wide ability to predict the data set with longer occurrence^47^. Differently from traditional linear regression models, ANN processes large datasets and still allows for an eventual prediction through a single output signal^48^. This neuron represented, in this case, a variable of easy measurement monitored during the established time of grain transport. This predictive model made it possible to make decisions more assertively about the MAE^49^.

Regarding the electrical conductivity (EC), the M5P, and MLR algorithms outperformed the other models. For the M5P, there was a correlation of 0.93 and MAE OF 0.38, with R^2^ of 86.49% (Table 1) (Fig. 6D). Grains transported with high moisture contents suffered alterations at cellular levels by the intensification of the respiratory process of the grains^50^. The high electrical conductivity (EC) results occurred due to the rupture of the membrane and cell wall of the grains where the exudates were released and leached^51^. The damage to the cellular tissues also altered the physicochemical composition of the grains, especially the carbohydrates.

The M5P algorithm achieved the best results of r and MAE for predicting the electrical conductivity test in corn grains. Some studies comparing the use of algorithms to predict the changes in electrical conductivity in grains are still scarce in the literature. For this reason, M5P provided answers in a shorter time when compared to the traditional regression^38^. In the germination evaluation (GERM), the MLR, ANN, and M5P algorithms had the highest correlation coefficients (r) and lowest mean absolute errors (MAE) (Table 1 and Fig. 10C). However, applying the SK test (p < 0.05), the models that best predicted the germination results were the ANN and MLR algorithms (Fig. 2C). Grain germination (GERM) was susceptible to the effects of intergranular temperature and relative humidity, which indirectly interfered with moisture contents. This serial reaction impaired vigor, which consequently reduced grain germination^52,53^. However, from real-time monitoring of easily measured variables, grain germination can be predicted. The results of r and MAE demonstrated that the ANN models satisfactorily predicted germination. Grain germination (GERM) was susceptible to the effects of intergranular temperature and relative humidity, which indirectly interfered with moisture contents. This serial reaction impaired vigor, which consequently reduced grain germination^52,53^. However, from real-time monitoring of easily measured variables, grain germination can be predicted. The results of r and MAE demonstrated that the ANN models satisfactorily predicted germination.

### Monitoring and predicting the quality of corn grains during drying

**Figure 11:**
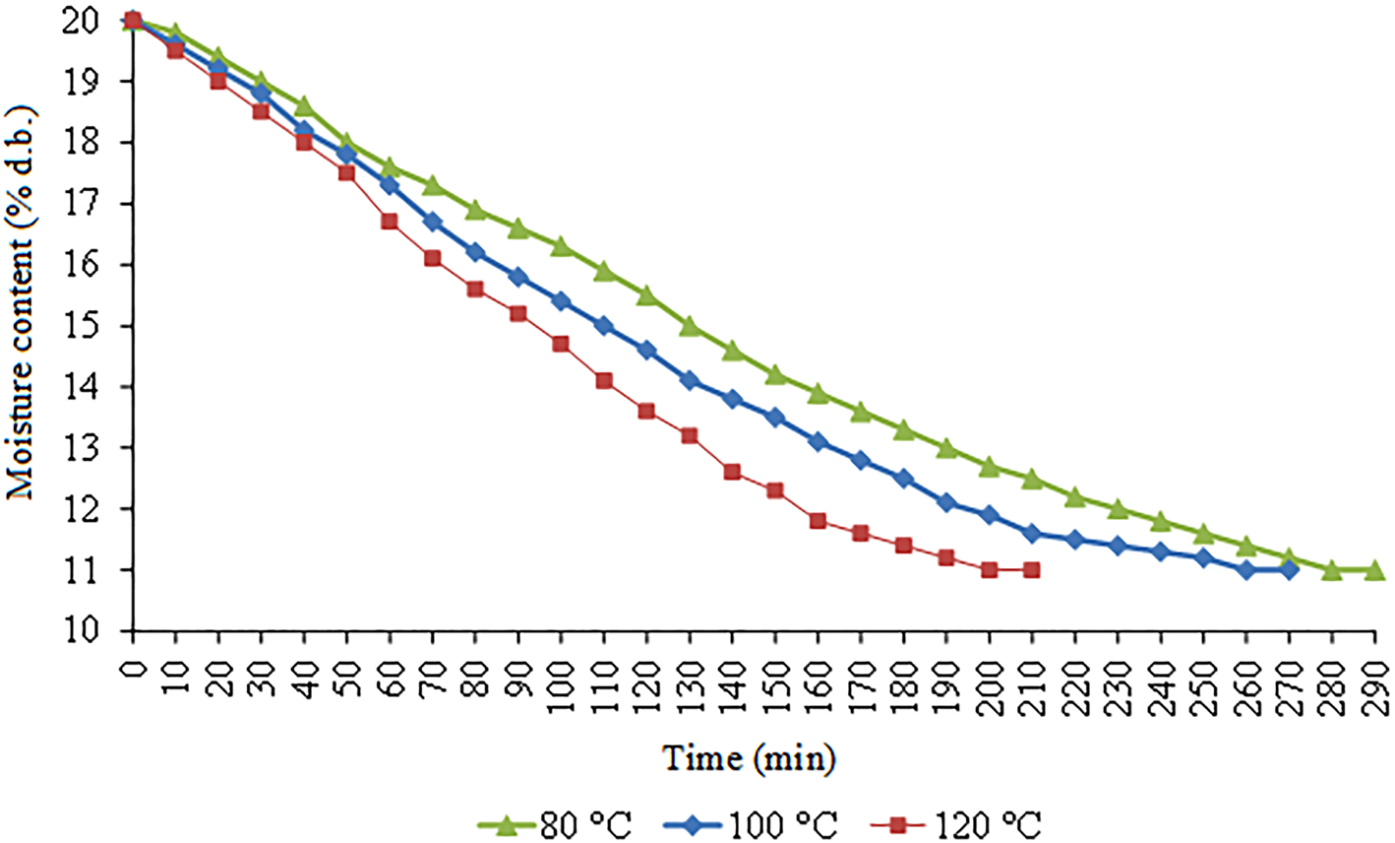
Drying curves of corn grains for different air temperatures.

Figure 11 shows the drying curves of corn grains for different drying air temperatures. We observed that the drying temperature of 80 °C extended the drying time to 4.83 h. Whereas, at a drying temperature of 100 °C, the time was reduced to 4.5, while the drying time was only 3.5 h at 120 °C. The grains reduced the moisture content by up to 11% for all drying conditions.

The difference in drying time of 1.83 h from 80 °C to 120 °C temperature can be attributed to the drying speed and higher grain mass flow in the processes preceding the drying. Increasing the drying air temperature from 80 °C to 120 °C can also affect the morphological structure of the grain and alter the cellular tissues, causing changes in starch, protein, and lipids. These findings were monitored and predicted to determine the best ML model to perform process control based on air temperature and drying time^54–56^.

**Figure 12:**
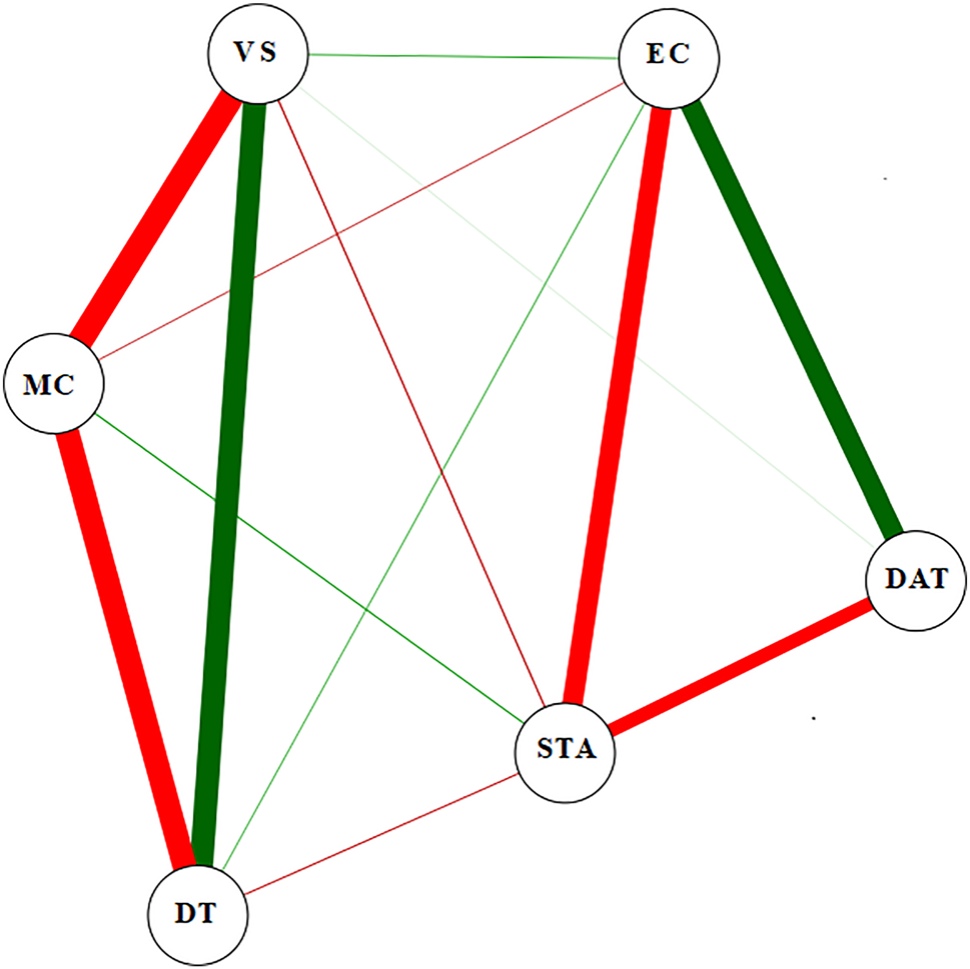
Pearson correlation network established between the variables: volumetric shrinkage (VS), starch (STA), electrical conductivity (EC), drying air temperature (DTA), moisture content (MC), and drying time (DT).

Figure 12 presents the correlations of the monitored and predicted variables. Drying temperature (DAT) was strongly and positively correlated with electrical conductivity (EC), while EC was positively and weakly correlated with volumetric shrinkage (VS). Ec had a strong and negative correlation with the grain starch yield (STA), while STA had a negative and moderate correlation with DAT. DT correlated negatively and strongly with moisture content (MC) and positively and strongly with VS, while VS had a strong and negative correlation with MC. There was a negative and weak correlation between DT x STA, VS x STA, MC x EC, and a weak and positive correlation between DT x EC and MC x STA.

**Figure 13:**
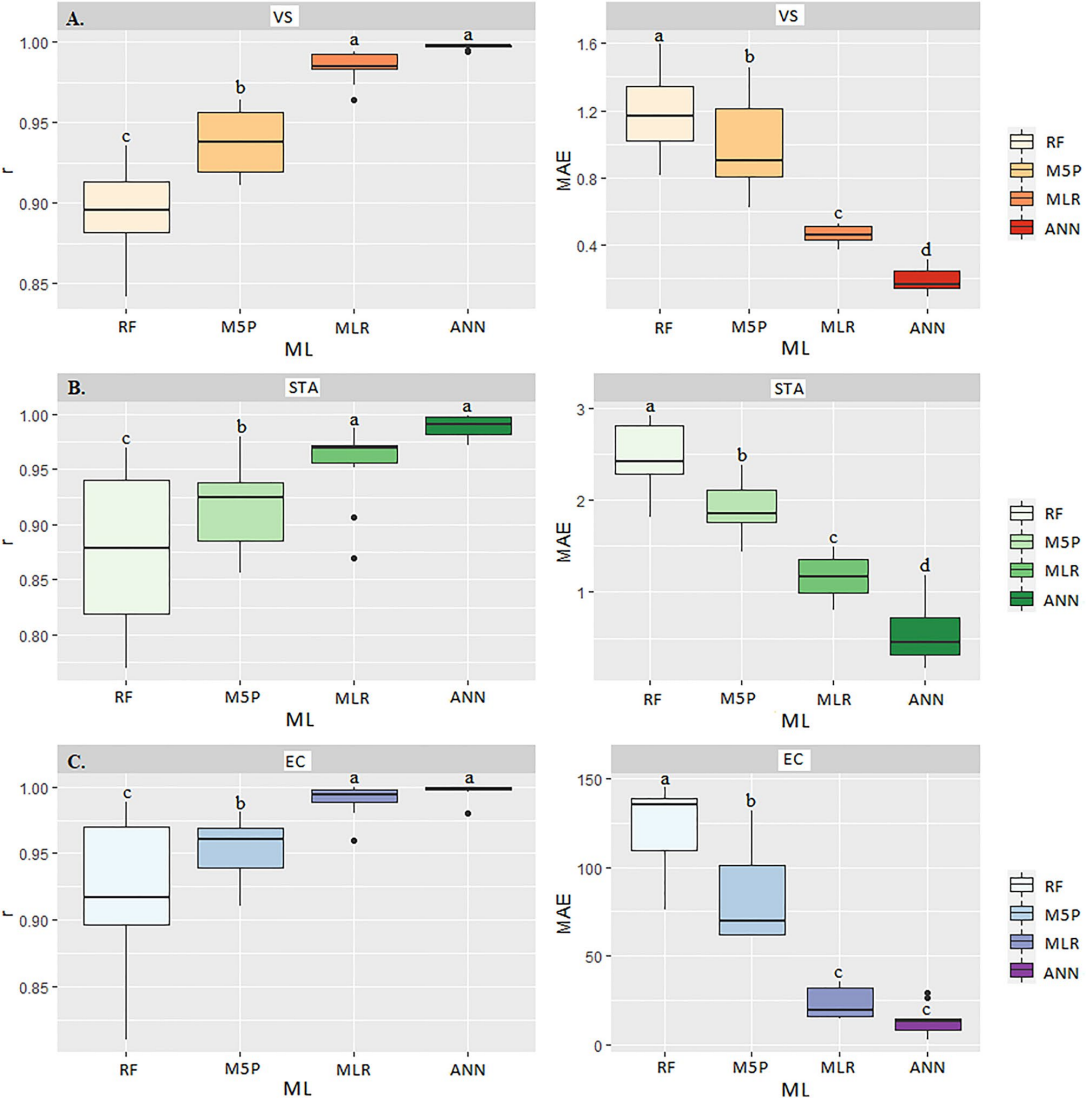
Boxplot for means comparison of correlation coefficient (r) and mean absolute error (MAE) between the multiple linear regression (MLR) and Machine Learning models: Artificial Neural Networks (ANN), M5P Algorithm (M5P), and Random Forest (RF) in predicting volumetric shrinkage-VS (**A**), starch-STA (**B**) and electrical conductivity-EC (**C**) in corn grains at drying stage.

Table 2 shows the results of correlation coefficients (r), coefficients of determination (R^2^), and mean absolute error (MAE) for predicting the corn grain quality variables at drying: volumetric shrinkage (VS), starch (STA), and electrical conductivity (EC). There were significant differences (p < 0.05) by SK test considering the different Machine Learning (ML) models and the monitored variables of drying air temperature (DTA), moisture content (MC), and drying time (DTA). Artificial neural network (ANN) obtained the highest r and lowest MAE and, therefore, is the most indicated model for predicting the variables VS, STA and EC (Table 2). In predicting the variables VS, STA and EC, the ANN model showed the highest r correlations (0.99, 0.98, and 0.99, respectively), but did not differ from the MLR by the SK test (p < 0.05) (Fig. 13A). The ANN model also showed the lowest mean MAE (0.20 and 0.52, respectively).

**Table 2:** Correlation coefficient (r), mean absolute error (MAE), and coefficient of determination (R^2^) between the observed and estimated values of dry matter loss, apparent mass specific, germination, and electrical conductivity of corn grain in the transport operation for the different Machine Learning models.

Models	r	MAE	R^2^	r	MAE	R^2^	r	MAE	R^2^
VS				STA			EC		
MLR	0.98a	0.46c	96.04	0.94a	1.14c	88.36	0.98a	21.98c	96.04
ANN	0.99a	0.20d	98.01	0.98a	0.52d	96.04	0.99a	13.21c	98.01
M5P	0.93b	0.97b	86.49	0.91b	1.84b	82.81	0.93b	81.68b	87.89
RF	0.88c	1.88a	77.44	0.86c	2.32a	73.96	0.89c	117.9a	80.96

*Equal letters in the column do not differ at p < 0.05 by the Scott Knott test. Dry Matter Loss (DML), Volumetric Shrinkage (VS), Starch (STA), and Electrical Conductivity (EC). Multiple Linear Regression (MLR), Artificial Neural Networks (ANN), Quinlan's M5 Algorithm (M5P), and Random Forest (RF).

The changes in the volumetric shrinkage (VS) resulted from the increase in drying air temperature that may have caused changes in the endosperm of the grains from the reduction of moisture contents. Thus, during the drying process, protein properties and total carbohydrates may have changed. When subjected to elevated temperatures, the carbohydrate molecules may have broken down and transformed into less complex molecules, multiplied in the intercellular space. These physicochemical changes compromised the quality of the grains^50^.

In starch yield prediction (STA), the ANN and RF models showed the highest correlation coefficients r (0.98 and 0.94, respectively), not differing from each other by SK test (p < 0.05) (Fig. 13B). The mean absolute error (MAE) values for each model were 0.52 and 1.14, respectively, indicating the ANN models with R^2^ accuracy of 96.04% (Table 2). Starch is a biomolecular carbohydrate that comprises most of the structure of corn grains and requires its components to be in perfect arrangements (H, O_2_, and C). Associated with the other components, when the grain undergoes high drying temperatures, molecular structures are affected^50^. Drying at temperatures above 80 °C affected the starch structure and its constituents^57^. In studies with corn drying, Timm et al.^56^ found that air drying temperature below 80 °C considerably extended the drying time, but the physicochemical constitution, especially the starch, is preserved. Meanwhile, for electrical conductivity, ANN and MLR models showed the highest correlation values r (0.98 and 0.99, respectively) and lowest MAE (21.98 and 13.21, respectively), not differing from each other by SK test (p < 0.05) (Fig. 13C), with better accuracy of R^2^ (98.04%) for ANN (Table 2). The increase in CE was linked to the rise in drying air temperature. With a more intense moisture movement from the interior of the grains to the surface in the inner layers of the grains, the cell walls of the grain structure were affected, causing the rupture of their membranes. When this occurred, as a consequence, exudates were released, raising the electrical conductivity of the grains^58^.

### Monitoring and predicting the quality of stored corn grains

**Figure 14:**
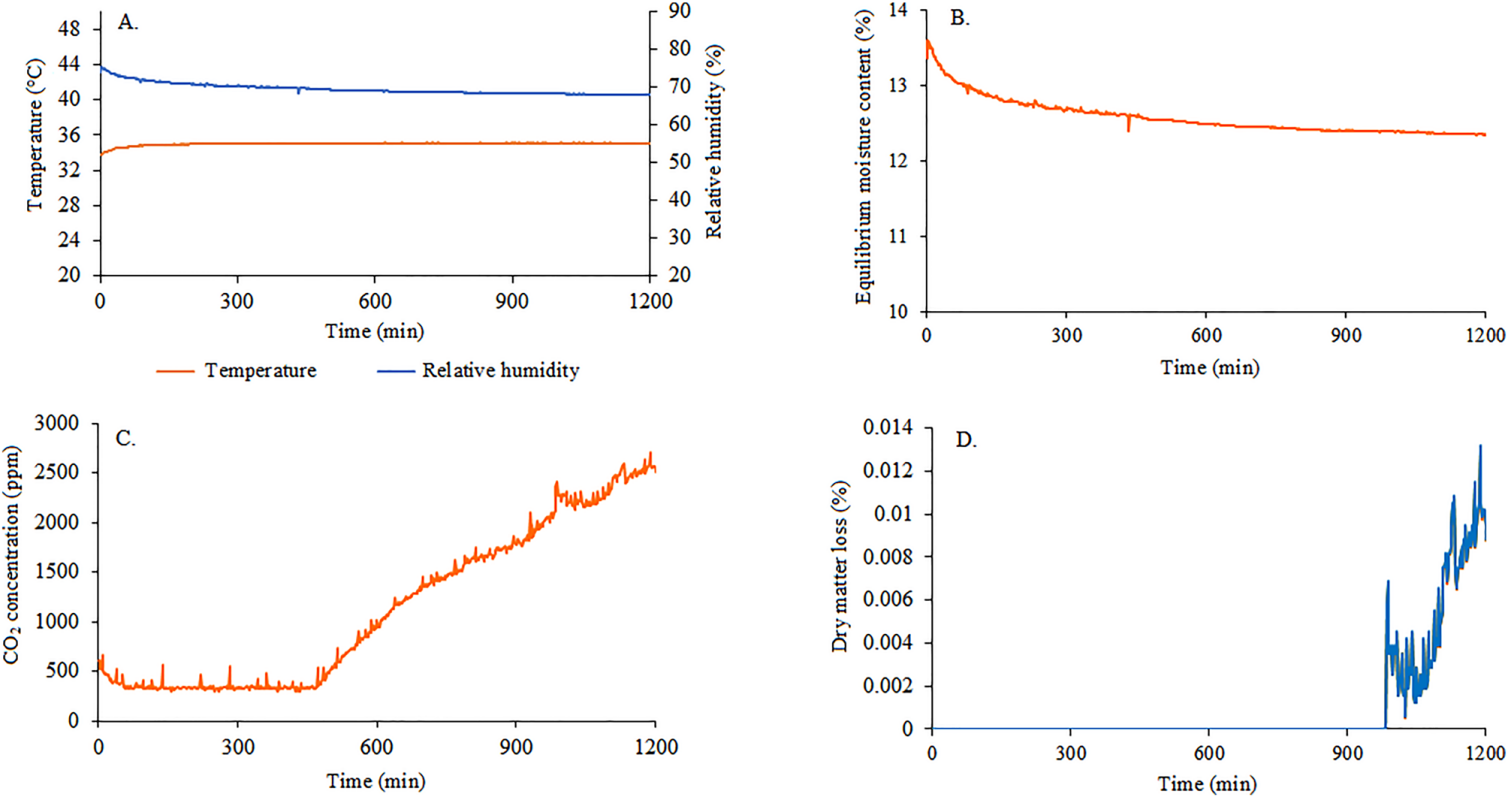
Early monitoring of relative humidity and intergranular temperature (**A**), equilibrium moisture content (**B**), CO_2_ concentrations (**C**), and dry matter loss (**D**), in corn grains at storage.

The results of temperature (T), intergranular relative humidity (RH) for calculation of equilibrium moisture content (EMC) of stored corn grain mass are shown in Fig. 14A,B. During the twenty-four hours of monitoring, temperature remained constant but high, between 30 and 36 °C, while the (RH) remained close to 70%, reaching 13% of EMC. From 7.5 h of monitoring, there was an increase in carbon dioxide (CO_2_) levels (Fig. 14C) and, consequently, higher dry matter consumption (Fig. 14D) at the end of the monitoring time (from 15 h on), following the peaks in grain respiration.

High temperatures, associated with high relative humidity, can trigger metabolic reactions in the grain^59^. During the release of these components, enzymes, and carbohydrates are degraded, reducing the quality of corn grains^14^. These relationships can be predicted and controlled to determine the optimal storage time of the grains without losses^20^.

**Figure 15:**
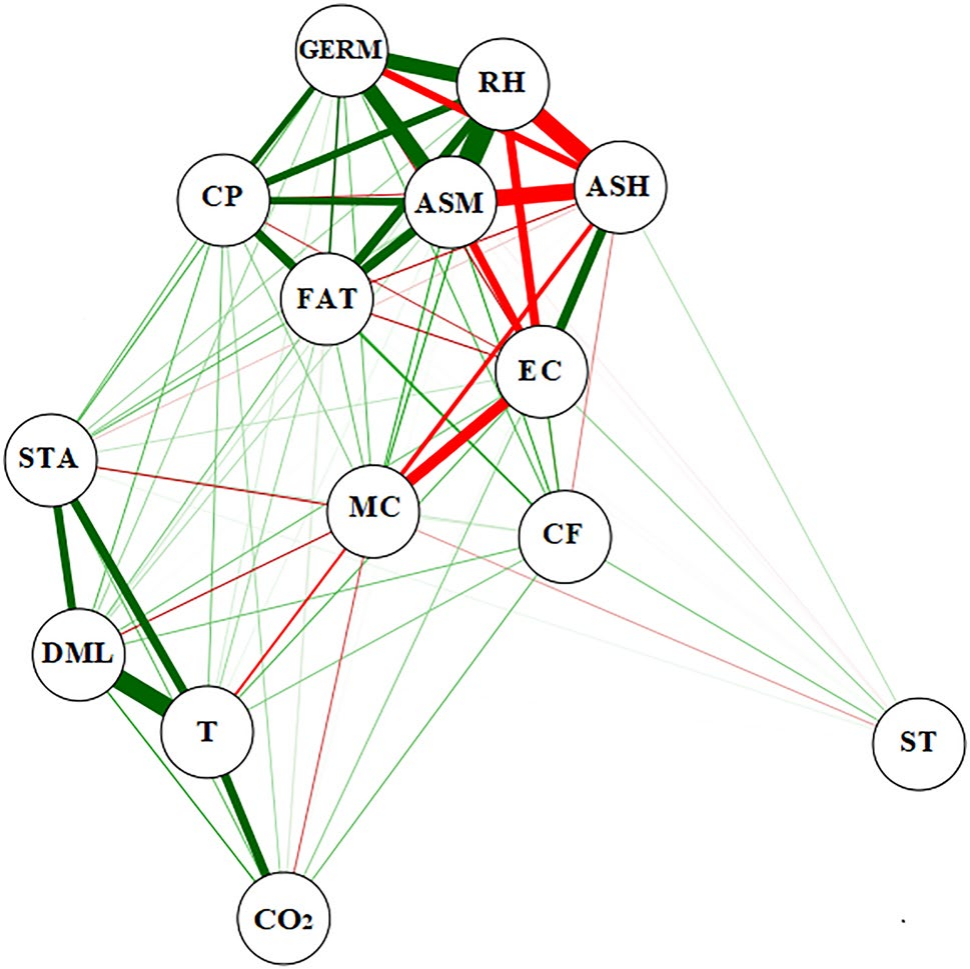
Pearson correlation network established between the variables: storage time (ST), intergranular temperature (T), intergranular relative humidity (RH), moisture content (MC), apparent specific mass (ASM), germination (GERM), electrical conductivity (EC), crude protein (CP), crude fiber (CF), fat (FAT), ash (ASH), starch (STA), carbon dioxide concentrations (CO_2_), and dry matter loss (DML).

Results of correlation between monitored and predicted variables are shown in Fig. 15. The variables RH and GERM had a high positive correlation between them and with ASM and medium positive correlation with CP. A medium positive correlation was also observed between FAT × ASM, GERM × CP, CP × ASM, CP × FAT, FAT × ASM, and EC x ASH. There was a high correlation between DML x T and a medium correlation between DML × STA, STA × T, and T × CO_2_. There was a high negative correlation between ASM × ASH, RH × ASH, and a medium negative correlation between GERM x ASH, ASM × EC, RH × EC, MC × EC. Furthermore, there was a weak but positive correlation between FAT × CF, PB × CF, CF × T, T × EC, T × ASH, CP × STA, CP × ST, GERM × ST, GERM × MC, GERM × T, CF × STA, DML × CO_2_, DML × CP, DML × FAT, DML × RH, DML × ASM, DML × EC, CO_2_ × STA, CO_2_ × CP, CO_2_ × FAT, and CO_2_ × CF. Weak and negative correlations were identified for ST × MC, STA × ST, T × MC, CP × EC, GERM × EC, CF × ASH, FAT v ASH, CP × ASH, and STA × ASH. It is noteworthy that among the monitored variables, RH had the most significant influence on grain quality^60,61^. Whereas, among the variables measured, ASM and GERM indicated positive and negative quality levels^62,63^.

Table 3 shows the results of the correlation coefficients (r), coefficient of determination (R^2^) and the mean absolute error (MAE) of the ML models for predicting the quality variables of stored corn grains: apparent specific mass (ASM), germination (GERM), electrical conductivity (EC), crude protein (CP), moisture content (MC), fats (FAT), crude fiber (CF), ash (ASH) and starch (STA) contents. The easy-to-measure input variables (T, RH, and ST) for the different ML models were significant (p < 0.05) by the Scott Knott (SK) test. The artificial neural network (ANN) and random forest (RF) models were the best predictors of MC, GERM, CP, CF, ASH, and FAT. Whereas the M5P model satisfactorily predicted ASM, EC, and STA.

**Table 3:** Correlation coefficient (r), mean absolute error (MAE), and coefficient of determination (R^2^) between the observed and estimated values of moisture content, apparent mass specific, electrical conductivity, germination, fat, ashes, starch, crude protein, and crude fiber of corn grain in the transport operation for the different Machine Learning models.

Models	r	MAE	R^2^	r	MAE	R^2^	r	MAE	R^2^
	MC			ASM			EC		
MLR	0.84c	0.89a	70.56	0.93b	2.69a	86.49	0.65b	96.93a	42.25
ANN	0.96a	0.45c	92.16	0.98a	1.43b	96.04	0.92a	54.75b	84.64
M5P	0.93b	0.53b	86.49	0.97a	1.59b	94.09	0.91a	57.33b	82.81
RF	0.97a	0.39c	94.09	0.98a	1.24b	96.04	0.94a	46.41b	88.36

Models	r	MAE	R^2^	r	MAE	R^2^	r	MAE	R^2^
	GERM			FAT			ASH		
MLR	0.75c	6.74a	56.25	0.69a	0.15a	47.61	0.90a	0.05a	81.00
ANN	0.94a	3.45b	88.36	0.73a	0.14a	53.29	0.90a	0.05a	81.00
M5P	0.89b	4.67c	79.21	0.71a	0.14a	50.41	0.91a	0.05a	82.81
RF	0.95a	3.13c	90.25	0.73a	0.14a	53.29	0.90a	0.05a	81.00

Models	r	MAE	R^2^	r	MAE	R^2^	r	MAE	R^2^
	STA			CP			CF		
MLR	0.68b	1.01a	46.24	0.75a	0.24a	56.25	0.56a	0.13a	31.36
ANN	0.88a	0.66b	77.44	0.76a	0.24a	57.76	0.62a	0.13a	38.44
M5P	0.87a	0.67b	75.69	0.77a	0.23a	59.29	0.59a	0.12a	34.81
RF	0.89a	0.63b	79.21	0.78a	0.23a	60.84	0.61a	0.13a	37.21

*Equal letters in the column do not differ at p < 0.05 by the Scott Knott test. Moisture Content (MC), Apparent Specific Mass (ASM), Electrical Conductivity (EC), Germination (GERM), FAT (FAT), Ash (ASH), Starch (STA), Crude Protein (CP), Crude Fiber (CF). Multiple Linear Regression (MLR), Artificial Neural Networks (ANN), Quinlan's M5 Algorithm (M5P), and Random Forest (RF).

**Figure 16:**
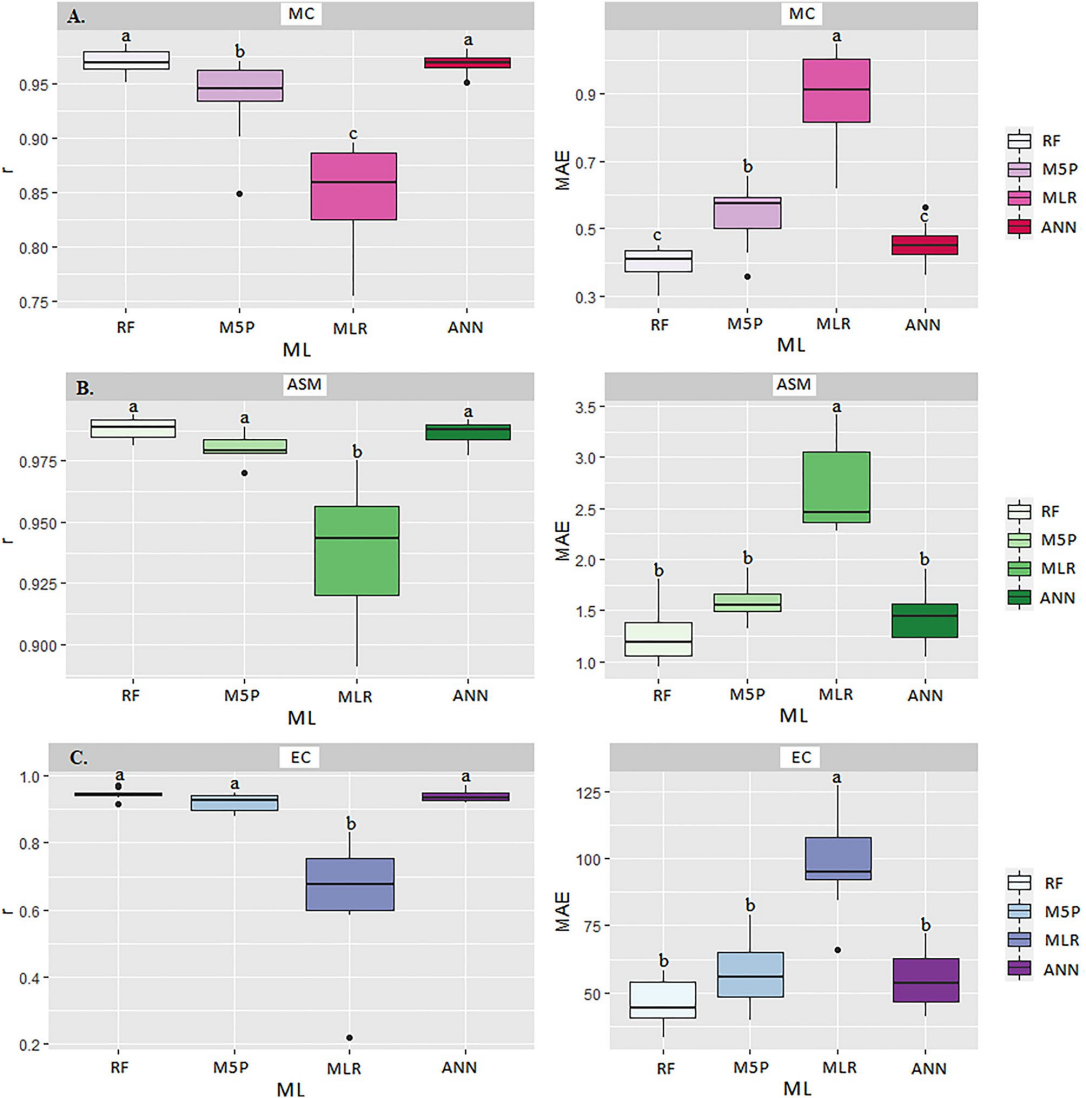
Boxplot for means comparison of correlation coefficient (r) and mean absolute error (MAE) between the multiple linear regression (MLR) and Machine Learning models: Artificial Neural Networks (ANN), M5P Algorithm (M5P), and Random Forest (RF) in predicting moisture content-MC (**A**), apparent specific mass-ASM (**B**) and electrical conductivity-EC (**C**) in corn grains at storage stage.

The ANN and RF models stood out in predicting the MC variable, reaching high r values (0.96 and 0.97, respectively), but not differing from each other by the SK test (p < 0.05) (Table 3 and Fig. 16A). The lower MAE means (0.45 and 0.39) allowed a better fit of the observed and predicted data, differentiating them from the M5P and MLR models, since these, even presenting relatively high r (0.84 and 0.93, respectively), showed a higher the MAE (0.89 and 0.53, respectively).

Even corn grains stored with a moisture content between 12 and 13% are susceptible to quality variation, depending on the conditions established in the grain mass. Storage conditions associated with the humidity and temperature are related to the water reabsorption in the grain. The grain mass's biological activity is more intense at high moisture content. In these cases, there is an increase in cellular respiration, besides the opportunity for pathogens to develop in the corn grains^64–66^. Furthermore, the moisture content in the grain mass can come from steps before storage, such as in low-efficiency drying, allowing the grain to enter into equilibrium moisture content with high moisture contents without being in optimal storage conditions^67^.

ANN and RF models were the best predictors of stored grain control. When trained, the ANNs efficiently predicted corn grains' physical and chemical quality during storage^21^. A study carried out by Córdova-Noboa et al.^11^ reinforces these findings, where stored corn grains dried at 35 °C obtained higher moisture contents (14.45%) over those dried at 120 °C (11.20%).

The ANN, M5P and RF models were superior in predicting the ASM variable, showing the highest r values (0.98, 0.97 and 0.98, respectively), without differing from each other by the SK test (p < 0.05) (Fig. 16B). The lowest r means (0.93) were observed for the traditional regression model, which consequently had the higher MAE (2.69) (Table 3).

Our findings indicated that ASM of corn grain were influenced by relative humidity and storage temperature. Furthermore, time was a factor that interfered with the specific mass of the stored grain^68,69^. Respiratory activity consumes dry matter and alters the properties of the grain mass, especially when temperature and moisture contents are high, boosting the process. Some authors have reported that, during serial reactions in the grain mass, oxidations of grain constituents occur, which consequently leads to losses of total carbohydrates, starch, proteins, and oils^70^. In research on grains stored in different packages, André et al.^25^ found that the ANN, M5P and RF models can be used to predict the apparent specific mass, supporting our results.

Even though the ANN, M5P, and RF models did not show significant differences among themselves by the SK test (p < 0.05) (Fig. 16C), they obtained the highest r values (0.92, 0.91, and 0.94, respectively) for CE. The high correlation was defined by the following MAE values (54.75, 57.33, and 46.41, respectively), while the random forests algorithm (RF) obtained the highest R^2^ (88.36%). Temperature, time, and moisture content influenced the increased electrical conductivity^44,70^. The contribution of these factors favored adverse reactions to grain quality, causing damage to the cell membrane^44,45^, which were best predicted by the RF, ANN, and M5P models.

**Figure 17:**
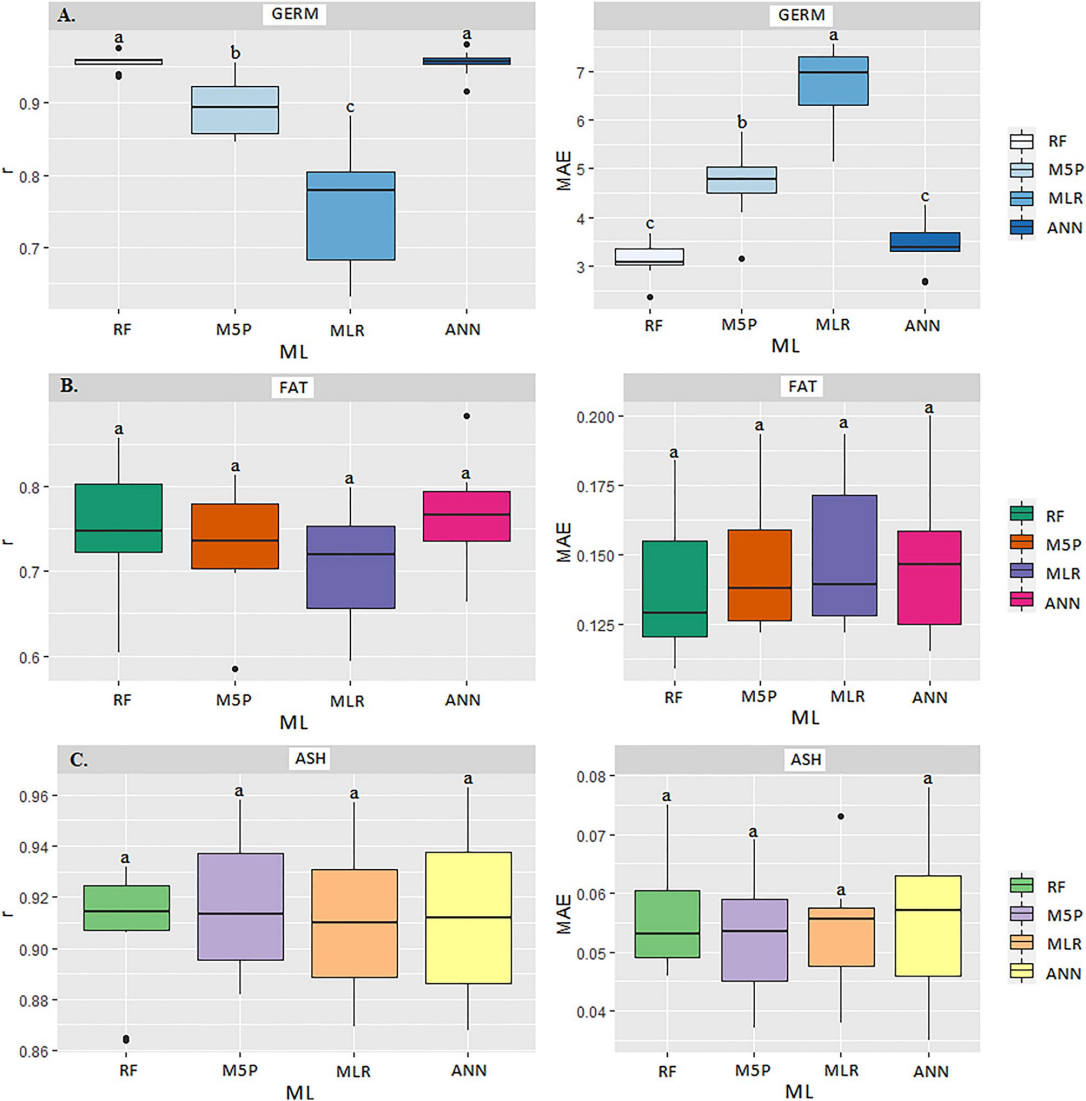
Boxplot for means comparison of correlation coefficient (r) and mean absolute error (MAE) between the multiple linear regression (MLR) and Machine Learning models: Artificial Neural Networks (ANN), M5P Algorithm (M5P), and Random Forest (RF) in predicting germination-GERM (**A**), fat-FAT (**B**) and ash-ASH (**C**) contents in corn grains at storage stage.

Regarding the germination variable (GERM), the ANN and RF models obtained the highest r correlations (0.94 and 0.95, respectively), not differing from each other by the SK test (p < 0.05) (Fig. 17A). There was a higher accuracy between monitored and predicted variables by the RF model, with R^2^ of 90.25% and lower MAE values (3.45 and 3.13, respectively) (Table 3). The MLR and M5P techniques were less accurate in predicting GERM, showing higher MAE (6.74 and 4.47, respectively) and r values (0.75 and 0.89).

Storage time and storage conditions accelerated biochemical reactions in the grain, degrading protein reserves, carbohydrates and negatively impacting germination^71,72^. We observed that high moisture content, relative humidity, temperature, and storage time increased oxidations and deteriorations in the embryo, reducing germination^73^. RF and ANN models were able to predict the germination outcomes accurately, supporting the findings reported by Zeymer et al.^43^.

There was no statistical difference among the models evaluated for the FAT variable. However, analyzing each ML model separately, the ANN and RF models showed the best results for r (0.73 and 0.73, respectively) and MAE (0.14) (Fig. 17B). Likewise, regarding the ASH variable, there was also no statistical difference by SK test (p < 0.05) between ML models (Fig. 17C). However, taking into account the higher r values (0.91), the M5P obtained the most accurate prediction, with R^2^ of 82.81% (Table 3).

**Figure 18:**
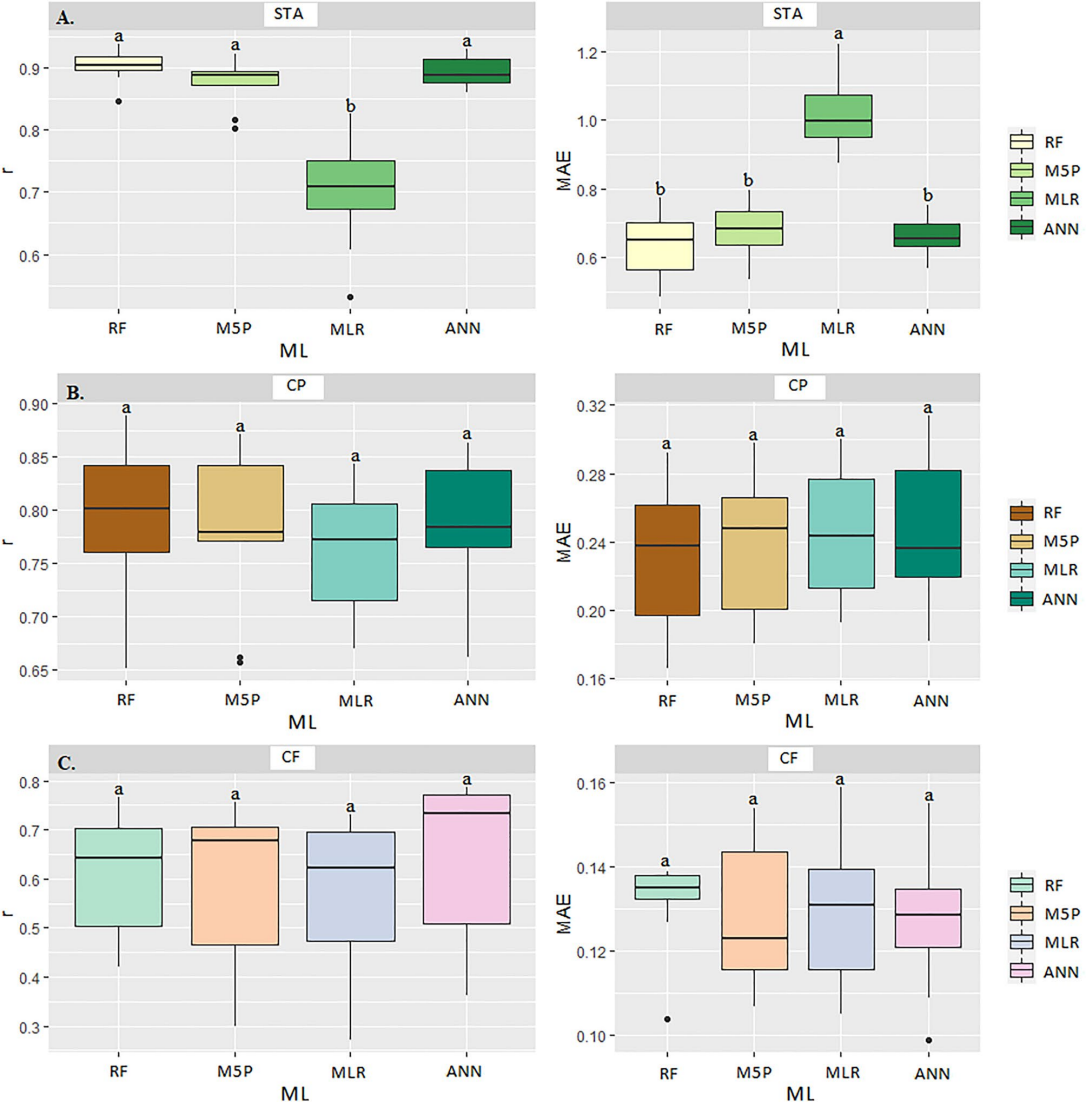
Boxplot for means comparison of correlation coefficient (r) and mean absolute error (MAE) between the multiple linear regression (MLR) and Machine Learning models: Artificial Neural Networks (ANN), M5P Algorithm (M5P), and Random Forest (RF) in predicting starch-STA (**A**), crude protein-CP (**B**) and crude fiber-CF (**C**) in corn grains at storage stage.

For starch (STA) prediction, the ANN, M5P, and RF models were the most accurate, which did not differ from each other by SK test (p < 0.05) (Fig. 18A). The traditional MLR model obtained the lowest accuracy, with the highest MAE (1.01) and lowest r value (0.68). It is also noteworthy that the model with the highest accuracy was RF, with R^2^ of 79.21%.

In the endosperm of corn grain, the average starch content is between 72.4 and 88%, corresponding to 83% of the dry grain mass^58^. As a carbohydrate, starch has a signaling function, regulating metabolic processes and stabilizing the cellular osmotic potential^74–77^. As a biochemical property, starch is vulnerable to the effects of storage conditions, especially grain mass temperature^65^.

Some studies have reported that corn stored for three months at temperatures below 20 °C maintained starch properties^77^. However, when high temperatures were checked, a reduction in corn starch contents and degradation was observed as early as 60 days^78^. Furthermore, corn storage technology may also contribute to reducing starch in corn. Studies find a reduction in total sugar contents in corn grain as a function of reduced starch contents when stored in paper packaging for 90 days^78^.

For reducing and estimating starch quality losses and assisting in decision making, the ANN, M5P and RF models obtained the best performance (Table 3 and Fig. 18A). For the crude protein (CP), there was no statistical difference among the models evaluated by the SK test (p < 0.05) (Fig. 18B). However, the random forest (RF) model presented the highest r (0.78) and lowest mean apparent error MAE (0.23), achieving an R^2^ of 60.84% (Table 3). There was no statistical difference among all models evaluated (p < 0.05) by the SK test for CF (Fig. 18C). For this variable, a satisfactory correlation between monitored and predicted data could not be found (Table 3).

The variables CP, FAT, CF and ASH were accurately predicted by RF, M5P, and ANN, with no statistical difference between the models. In a study by Alvarez et al.^78^, the authors verified that storage conditions influenced crude protein contents in corn grain mass, reducing to 10.6% in the 60-day storage period at a temperature of 16 °C. The quality of the stored grains is affected by elevating the moisture contents of the corn grain mass^61^. Analyzing some of these biochemical properties in corn grains stored at 180 days, Queiroz et al.^79^ found that ash and crude fiber contents increased from 1.27% to 1.45% and reduced from 11.1 to 9.5%, respectively, with no changes in lipids (4.7%).

## Conclusion

**Figure 19:**
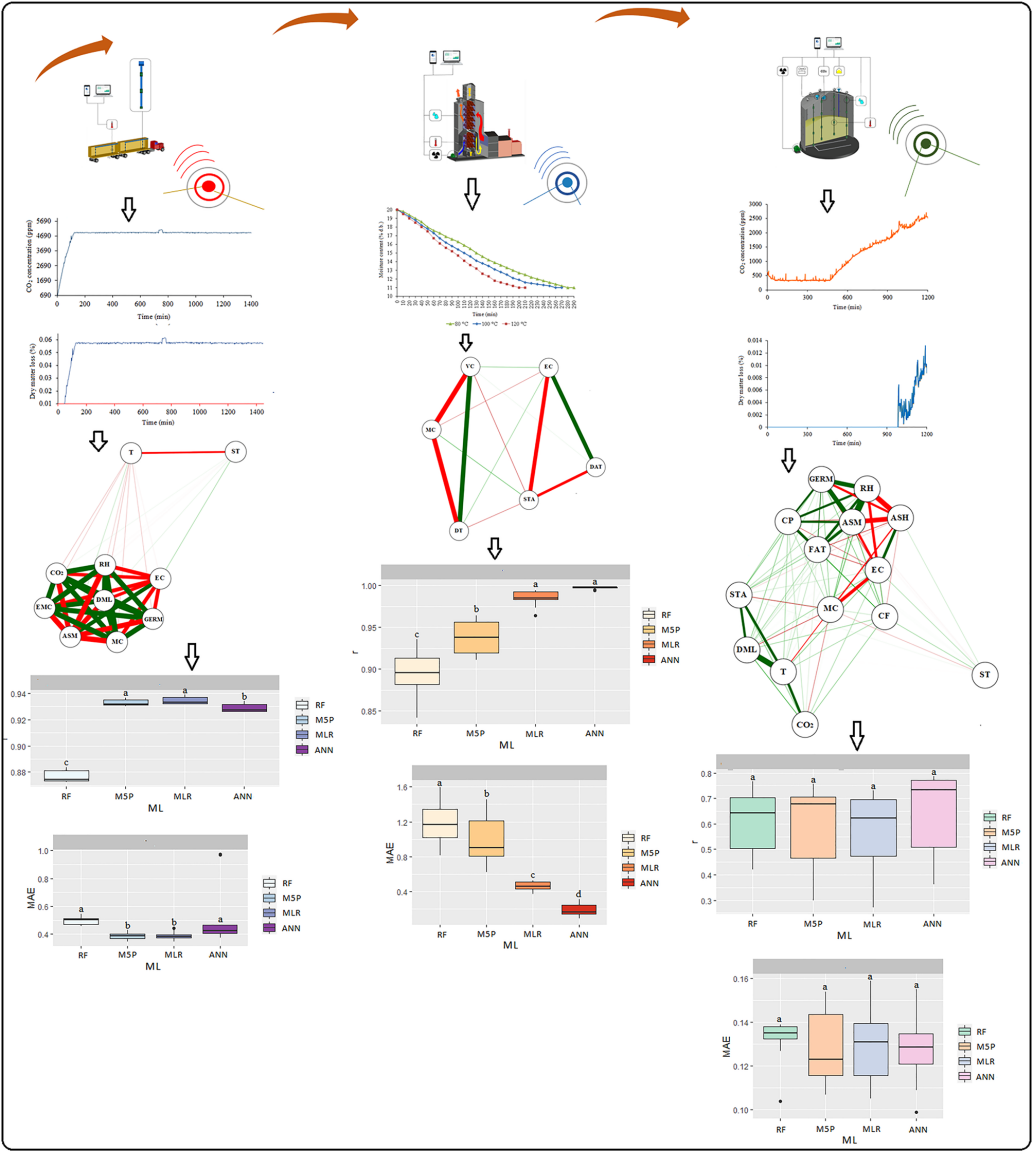
Synthesis of the results and monitoring and prediction scheme for post-harvest processes.

Advances in grain quality monitoring technologies in post-harvest processes are being opened with the application of artificial intelligence. These advances support strategies to prevent post-harvest grain spoilage. In this study, it was concluded that the corn grain quality at the different post-harvest stages was satisfactorily predicted by the Machine Learning models. At grain transport, the ANN, M5P, and RF models obtained the best prediction results for dry matter loss, apparent specific mass, electrical conductivity, and germination. At drying stage, the ANN and RF models are the best predictors of starch yield, volumetric shrinkage, and electrical conductivity. At storage, the ANN and RF models are suitable for predicting the moisture content and germination variables. The M5P model successfully predicted the bulk density, electrical conductivity, and germination. We highlight the RF model and ANN as the most suitable for predicting corn grain quality at different post-harvest stages due to its simplicity, processing speed, and ability to reveal the levels of importance of the variables that best contribute to the model fit. From this, it is suggested the application of sensors for real-time monitoring of easily measured variables makes it possible to more adequately control post-harvest processes (Fig. 19), as well as indirectly predict grain quality losses through machine learning models.

### Supplementary Information

Supplementary Table S1.

Supplementary Table S2.

Supplementary Table S3.

## Data Availability

The author (Paulo Carteri Coradi—paulocoradi@yahoo.com.br) make available the research data and materials.
