# Supplementary material for: Monitoring and predicting corn grain quality on the transport and post-harvest operations in storage units using sensors and machine learning models
Source: Sci Rep. 2024 Mar 14;14:6232. doi: 10.1038/s41598-024-56879-5 (PMC10940695; doi:10.1038/s41598-024-56879-5)
Supplement: Supplementary file 1 — Supplementary Table S1. [file 41598_2024_56879_MOESM1_ESM.doc]

**Table S1**. Parameters used to predict quality variables in the corn grain transport stage

| Models | Parameters |
| --- | --- |
|  | **Electrical Conductivity (EC)** |
| MLR | weka.classifiers.functions.LinearRegression -S 0 -R 1.0E-8 -num-decimal-places 4 (Cross-validation folds 10) |
| ANN | weka.classifiers.functions.MultilayerPerceptron -L 0.01 -M 0.01 -N 3500 -V 0 -S 1 -E 20 -H "20, 20" -output-debug-info (Cross-validation folds 10) |
| M5P | weka.classifiers.trees.M5P-M 4.0 -num-decimal-places 4 (Cross-validation folds 10) |
| RF | weka.classifiers.trees.RandomForest -P 100 -I 400 -num-slots 1 -K 1 -M 1.0 -V 0.001 -S 2 -output-debug-info (Cross-validation folds 10) |
|  | **Apparent Specific Mass (ASM)** |
| MLR | weka.classifiers.functions.LinearRegression -S 0 -R 1.0E-8 -num-decimal-places 4 (Cross-validation- folds 10) |
| ANN | weka.classifiers.functions.MultilayerPerceptron -L 0.3 -M 0.2 -N 500 -V 0 -S 0 -E 20 -H "2, 2" -output-debug-info (Cross-validation-folds 10) |
| M5P | weka.classifiers.trees.M5P -R -M 4.0 -output-debug-info -num-decimal-places 4 (Cross-validation-folds 10) |
| RF | weka.classifiers.trees.RandomForest -P 100 -I 500 -num-slots 1 -K 0 -M 1.0 -V 0.001 -S 1 -output-debug-info (Cross-validation-folds 10) |
|  | **Dry Matter Loss (DML)** |
| MLR | weka.classifiers.functions.LinearRegression -S 0 -R 1.0E-8 -num-decimal-places 4 (Cross-validation-folds 10) |
| ANN | weka.classifiers.functions.MultilayerPerceptron -L 0.3 -M 0.2 -N 500 -V 0 -S 0 -E 20 -H "2, 4" (Cross-validation-folds 10) |
| M5P | weka.classifiers.trees.M5P -N -M 4.0 -output-debug-info -num-decimal-places 4 (Cross-validation-folds 10) |
| RF | weka.classifiers.trees.RandomForest -P 100 -attribute-importance -I 500 -num-slots 1 -K 0 -M 1.0 -V 0.001 -S 1 (Cross-validation-folds 10) |
|  | **Germination (GERM)** |
| MLR | weka.classifiers.functions.LinearRegression -S 0 -R 1.0E-8 -num-decimal-places 4 (Cross-validation-folds 10) |
| ANN | weka.classifiers.functions.MultilayerPerceptron -L 0.1 -M 0.01 -N 500 -V 0 -S 0 -E 20 -H "1, 1" -G -R -output-debug-info (Cross-validation-folds 10) |
| M5P | weka.classifiers.trees.M5P -R -M 4.0 -output-debug-info -num-decimal-places 4 (Cross-validation-folds 10) |
| RF | weka.classifiers.trees.RandomForest -P 100 -attribute-importance -I 100 -num-slots 1 -K 0 -M 1.0 -V 0.001 -S 1 -output-debug-info (Cross-validation-folds 10) |
