# Supplementary material for: Monitoring and predicting corn grain quality on the transport and post-harvest operations in storage units using sensors and machine learning models
Source: Sci Rep. 2024 Mar 14;14:6232. doi: 10.1038/s41598-024-56879-5 (PMC10940695; doi:10.1038/s41598-024-56879-5)
Supplement: Supplementary file 2 — Supplementary Table S2. [file 41598_2024_56879_MOESM2_ESM.doc]

**Table S2**. Parameters used to predict the quality variables in the corn grain drying stage

| Models | Parameters |
| --- | --- |
|  | **Electric Conductivity (EC)** |
| MLR | weka.classifiers.functions.LinearRegression -S 0 -R 1.0E-8 -num-decimal-places 4- cross-validation-fold 10 |
| ANN | weka.classifiers.functions.MultilayerPerceptron -L 0.3 -M 0.2 -N 500 -V 0 -S 0 -E 20 -H a- cross-validation-fold 10 |
| M5P | weka.classifiers.trees.M5P -R -M 4.0 -output-debug-info -num-decimal-places 4- cross-validation-fold 10 |
| RF | weka.classifiers.trees.RandomForest -P 100 -attribute-importance -I 100 -num-slots 1 -K 0 -M 1.0 -V 0.001 -S 1 -depth 1 -output-debug-info- cross-validation-fold 10 |
|  | **Volumetric Shrinkage (VS)** |
| MLR | weka.classifiers.functions.LinearRegression -S 0 -R 1.0E-8 -output-debug-info -num-decimal-places 4- cross-validation-fold 10 |
| ANN | weka.classifiers.functions.MultilayerPerceptron -L 0.3 -M 0.2 -N 500 -V 0 -S 0 -E 20 -H "2, 2" -output-debug-info-cross-validation-fold 10 |
| M5P | weka.classifiers.trees.M5P -R -M 4.0 -output-debug-info -num-decimal-places 4- cross-validation-fold 10 |
| RF | weka.classifiers.trees.RandomForest -P 100 -attribute-importance -I 100 -num-slots 1 -K 0 -M 1.0 -V 0.001 -S 1 -depth 1 -output-debug-info- cross-validation-fold 10 |
|  | **Starch (STA)** |
| MLR | weka.classifiers.functions.LinearRegression -S 0 -R 1.0E-8 -output-debug-info -num-decimal-places 4- cross-validation-fold 10 |
| ANN | weka.classifiers.functions.MultilayerPerceptron -L 0.3 -M 0.2 -N 500 -V 0 -S 0 -E 20 -H "2, 2" -output-debug-info- cross-validation-fold 10 |
| M5P | weka.classifiers.trees.M5P -R -M 4.0 -output-debug-info -num-decimal-places 4- cross-validation-fold 10 |
| RF | weka.classifiers.trees.RandomForest -P 100 -I 100 -num-slots 1 -K 0 -M 1.0 -V 0.001 -S 1 -depth 1 -B -output-debug-info- cross-validation-fold 10 |
