# Supplementary material for: Monitoring and predicting corn grain quality on the transport and post-harvest operations in storage units using sensors and machine learning models
Source: Sci Rep. 2024 Mar 14;14:6232. doi: 10.1038/s41598-024-56879-5 (PMC10940695; doi:10.1038/s41598-024-56879-5)
Supplement: Supplementary file 3 — Supplementary Table S3. [file 41598_2024_56879_MOESM3_ESM.doc]

**Table S3.** Parameters used to predict the quality variables in the corn grain storage stage

| Models | Parameters |
| --- | --- |
|  | **Apparent Specific Mass (ASM)** |
| MLR | weka.classifiers.functions.LinearRegression -S 0 -R 1.0E-8 -output-debug-info -num-decimal-places 4-Cross-validation-fold 10 |
| ANN | weka.classifiers.functions.MultilayerPerceptron -L 0.1 -M 0.2 -N 2500 -V 0 -S 0 -E 20 -H "10, 10" -output-debug-info Cross-validation-fold 10 |
| M5P | weka.classifiers.trees.M5P -M 4.0 -output-debug-info -num-decimal-places 4 Cross-validation-fold 10 |
| RF | weka.classifiers.trees.RandomForest -P 100 -I 100 -num-slots 1 -K 0 -M 1.0 -V 0.001 -S 0 -output-debug-info-Cross-validation-fold 10 |
|  | **Germination (GERM)** |
| MLR | weka.classifiers.functions.LinearRegression -S 0 -R 1.0E-8 -output-debug-info -num-decimal-places 4- Cross-validation-fold 10 |
| ANN | weka.classifiers.functions.MultilayerPerceptron -L 0.3 -M 0.2 -N 1000 -V 0 -S 1 -E 20 -H "10, 10" -output-debug-info- Cross-validation-fold 10 |
| M5P | weka.classifiers.trees.M5P -M 4.0 -output-debug-info -num-decimal-places 4- Cross-validation-fold 10 |
| RF | weka.classifiers.trees.RandomForest -P 100 -I 100 -num-slots 1 -K 0 -M 1.0 -V 0.001 -S 1 -output-debug-info- Cross-validation-fold 10 |
|  | **Electric Conductivity (EC)** |
| MLR | weka.classifiers.functions.LinearRegression -S 0 -R 1.0E-8 -output-debug-info -num-decimal-places 4- Cross-validation-fold 10 |
| ANN | weka.classifiers.functions.MultilayerPerceptron -L 0.3 -M 0.2 -N 3500 -V 0 -S 0 -E 20 -H "10, 10" -output-debug-info- Cross-validation-fold 10 |
| M5P | weka.classifiers.trees.M5P -M 4.0 -output-debug-info -num-decimal-places 4- Cross-validation-fold 10 |
| RF | weka.classifiers.trees.RandomForest -P 100 -I 100 -num-slots 1 -K 0 -M 1.0 -V 0.001 -S 1 -output-debug-info- Cross-validation-fold 10 |
| **Crude Protein (CP)** | |
| MLR | weka.classifiers.functions.LinearRegression -S 0 -R 1.0E-8 -output-debug-info -num-decimal-places 4- Cross-validation-fold 10 |
| ANN | weka.classifiers.functions.MultilayerPerceptron -L 0.1 -M 0.2 -N 500 -V 0 -S 1 -E 20 -H "8, 8" -output-debug-info -num-decimal-places 4- Cross-validation-fold 10 |
| M5P | weka.classifiers.trees.M5P -M 4.0 -output-debug-info -num-decimal-places 4- Cross-validation-fold 10 |
| RF | weka.classifiers.trees.RandomForest -P 100 -I 500 -num-slots 1 -K 0 -M 1.0 -V 0.001 -S 0 -depth 4 -output-debug-info -num-decimal-places 6- Cross-validation-fold 10 |
| **Moisture Content (MC)** | |
| MLR | weka.classifiers.functions.LinearRegression -S 0 -R 1.0E-8 -num-decimal-places 4- Cross-validation-fold 10 |
| ANN | weka.classifiers.functions.MultilayerPerceptron -L 0.3 -M 0.1 -N 4500 -V 0 -S 1 -E 20 -H "10, 10" -output-debug-info -num-decimal-places 6- Cross-validation-fold 10 |
| M5P | weka.classifiers.trees.M5P -M 4.0 -output-debug-info -num-decimal-places 4- Cross-validation-fold 10 |
| RF | weka.classifiers.trees.RandomForest -P 100 -attribute-importance -I 100 -num-slots 1 -K 0 -M 1.0 -V 0.001 -S 1 -output-debug-info -num-decimal-places 4- Cross-validation-fold 10 |
| **Fat (FAT)** | |
| MLR | weka.classifiers.functions.LinearRegression -S 0 -R 1.0E-8 -output-debug-info -num-decimal-places 4- Cross-validation-fold 10 |
| ANN | weka.classifiers.functions.MultilayerPerceptron -L 0.1 -M 0.2 -N 2500 -V 0 -S 1 -E 20 -H "2, 2" -output-debug-info-Cross-validation-fold 10 |
| M5P | weka.classifiers.trees.M5P -N -R -M 4.0 -output-debug-info -num-decimal-places 6- Cross-validation-fold 10 |
| RF | weka.classifiers.trees.RandomForest -P 100 -attribute-importance -I 500 -num-slots 1 -K 0 -M 1.0 -V 0.001 -S 1 -depth 3 -output-debug-info -num-decimal-places 4- Cross-validation-fold 10 |
| **Crude Fiber (CF)** | |
| MLR | weka.classifiers.functions.LinearRegression -S 0 -R 1.0E-8 -output-debug-info -num-decimal-places 4- Cross-validation-fold 10 |
| ANN | weka.classifiers.functions.MultilayerPerceptron -L 0.2 -M 0.1 -N 500 -V 0 -S 1 -E 20 -H "25, 25" -output-debug-info- Cross-validation-fold 10 |
| M5P | weka.classifiers.trees.M5P -N -R -M 5.0 -output-debug-info -num-decimal-places 4- Cross-validation-fold 10 |
| RF | weka.classifiers.trees.RandomForest -P 100 -attribute-importance -I 200 -num-slots 1 -K 0 -M 1.0 -V 0.001 -S 1- Cross-validation-fold 10 |
| **Ashes (ASH)** | |
| MLR | weka.classifiers.functions.LinearRegression -S 0 -R 1.0E-8 -num-decimal-places 4-Cross-validation-fold 10 |
| ANN | weka.classifiers.functions.MultilayerPerceptron -L 0.1 -M 0.2 -N 2000 -V 0 -S 0 -E 20 -H "4, 4" -output-debug-info -num-decimal-places 4-Cross-validation-fold 10 |
| M5P | weka.classifiers.trees.M5P -N -M 2.0 -output-debug-info -num-decimal-places 4- Cross-validation-fold 10 |
| RF | weka.classifiers.trees.RandomForest -P 100 -I 400 -num-slots 1 -K 0 -M 1.0 -V 0.001 -S 4 -output-debug-info -num-decimal-places 4- Cross-validation-fold 10 |
| **Starch (STA)** | |
| MLR | weka.classifiers.functions.LinearRegression -S 0 -R 1.0E-8 -output-debug-info -num-decimal-places 4- Cross-validation-fold 10 |
| ANN | weka.classifiers.functions.MultilayerPerceptron -L 0.1 -M 0.2 -N 1000 -V 0 -S 0 -E 20 -H "8, 8" -output-debug-info -num-decimal-places 4- Cross-validation-fold 10 |
| M5P | weka.classifiers.trees.M5P -M 6.0 -output-debug-info -num-decimal-places 4- Cross-validation-fold 10 |
| RF | weka.classifiers.trees.RandomForest -P 100 -attribute-importance -I 300 -num-slots 1 -K 0 -M 1.0 -V 0.001 -S 2 -output-debug-info -num-decimal-places 6- Cross-validation-fold 10 |
